# New Insights on Biological Activities, Chemical Compositions, and Classifications of Marine Actinomycetes Antifouling Agents

**DOI:** 10.3390/microorganisms11102444

**Published:** 2023-09-29

**Authors:** Radwa N. Morgan, Amer Al Ali, Mohammad Y. Alshahrani, Khaled M. Aboshanab

**Affiliations:** 1 National Centre for Radiation Research and Technology (NCRRT), Drug Radiation Research Department, Egyptian Atomic Energy Authority (EAEA), Ahmed El-Zomor St, Cairo 11787, Egypt; radwa.morgan@eaea.sci.eg; 2 Department of Clinical Laboratory Sciences, Faculty of Applied Medical Sciences, University of Bisha, 255, Al Nakhil, Bisha 67714, Saudi Arabia; ameralali@ub.edu.sa; 3 Department of Clinical Laboratory Sciences, College of Applied Medical Sciences, King Khalid University, Abha 9088, Saudi Arabia; moyahya@kku.edu.sa; 4 Microbiology and Immunology Department, Faculty of Pharmacy, Ain Shams University, African Union Organization Street, Abbassia, Cairo 11566, Egypt

**Keywords:** marine actinomycetes, biofouling, antibacterial, antifouling, alkaloids, polyketide, terpenoids, quorom sensing inhibitors, antibiofilm

## Abstract

Biofouling is the assemblage of undesirable biological materials and macro-organisms (barnacles, mussels, etc.) on submerged surfaces, which has unfavorable impacts on the economy and maritime environments. Recently, research efforts have focused on isolating natural, eco-friendly antifouling agents to counteract the toxicities of synthetic antifouling agents. Marine actinomycetes produce a multitude of active metabolites, some of which acquire antifouling properties. These antifouling compounds have chemical structures that fall under the terpenoids, polyketides, furanones, and alkaloids chemical groups. These compounds demonstrate eminent antimicrobial vigor associated with antiquorum sensing and antibiofilm potentialities against both Gram-positive and -negative bacteria. They have also constrained larval settlements and the acetylcholinesterase enzyme, suggesting a strong anti-macrofouling activity. Despite their promising in vitro and in vivo biological activities, scaled-up production of natural antifouling agents retrieved from marine actinomycetes remains inapplicable and challenging. This might be attributed to their relatively low yield, the unreliability of in vitro tests, and the need for optimization before scaled-up manufacturing. This review will focus on some of the most recent marine actinomycete-derived antifouling agents, featuring their biological activities and chemical varieties after providing a quick overview of the disadvantages of fouling and commercially available synthetic antifouling agents. It will also offer different prospects of optimizations and analysis to scale up their industrial manufacturing for potential usage as antifouling coatings and antimicrobial and therapeutic agents.

## 1. Introduction

Biological fouling, often known as biofouling, is an engineering problem that plagues numerous industries. It is the buildup of undesired biological material, corrosions, and suspended particles on surfaces. Fouling varies greatly between medicinal, marine, and industrial applications with significantly high financial burdens [1]. Marine biofouling is also a pertaining issue that has been associated with diminished drill ship efficiencies, high fuel consumption rates among commercial tanks and vessels that consequently level up greenhouse gas emissions, and reared environmental pollution [2,3]. Additionally, ship and vessel biofouling can transfer non-native macro/microorganisms from one region to another, which may disrupt the local marine biodiversity and alter the ecological balance [3,4]. It has been a usual practice to coat the submerged surfaces of artificial structures (hulls, ships, pipelines) with antifouling paints that incorporate biocidal substances and to release them at a controlled rate to prevent unwelcome biofouling [5]. Ancient mariners used copper coats extensively to deter biofouling. However, Cu^2+^ ions unselectively attach to the acidic polysaccharide residues within the planktonic species cell wall, resulting in the oxidation of thiol-rich proteins and intracellular Cu^2+^ accumulation. Long-term use of copper as a marine antifouling biocide was connected to a range of toxicities, including growth inhibition, delayed gametophyte and sporophyte development, and spore discharge in a number of planktonic species [6,7].

The three main categories of antifouling paints currently in use are self-polishing systems, ablative paints, and soluble matrix paints, generally known as conventional paints [8]. Tributyltin (TBT) released from self-polishing copolymer (SPC) has been used instead of copper coats, and it successfully eliminated settling fouling organisms [9]. However, TBT is not selective; it affects off-target creatures and disturbs the ecosystem balance (disappearance of shellfish, invertebrate species; dog whelk, *Nucella lapillus*). For its toxic effects, TBT is prohibited in many regions [10]. Few other antifouling biocides are now in use; these include irgarol 1051 (a triazine herbicide), zinc pyrithione (an anti-dandruff fungicide), and Sea-Nine 211 (an isothiazolone). These are mostly employed with copper as co-biocides, particularly to boost the effectiveness against algae [11]. However, toxicities are reported from these antibiofouling coats [12], which necessitate the presence of environmentally benign and eco-friendly natural antifouling agents. Researchers gathered different antifouling compounds from different natural sources (e.g., microorganisms, sponges, plants, algae, etc.) that were more selective against fouling species and had lower toxicity [13,14]. 

Marine aquatic invertebrates, like sponges and corals, as well as vertebrates like pufferfish, are associated with marine actinomycetes [15]. Interestingly, not only can marine actinomycetes be isolated from marine sediments, but they can coexist with other species and live in both planktonic and biofilm habitats [16]. Most of the novel biologically active compounds (antibacterial, anticancer, antifouling, etc.) were discovered by studying marine actinomycetes, which have recently garnered a lot of interest. Different genera of actinomycetes, including *Streptomyces*, *Actinomyces*, *Arthrobacter*, *Corynebacterium*, and *Micromonospora*, can generate a variety of chemicals with a spacious spectrum of activities spanning various biological aspects [17]. Due to their ability to adapt to a variety of severe environmental factors (variable pH, temperature, salinity), marine actinomycetes secrete these biologically active substances in response to the surrounding stress inflicted upon them [17]. Marine actinomycetes are also rich sources of natural antifouling/antibacterial agents with EC50 values < 25 μg/mL [18]. According to the US Navy program, a safe, eco-friendly antifouling agent must record an EC50 < 25 μg/mL. The LC50 value is the 50% fatal concentration for the tested organism, whereas the EC50 value is the median effective concentration at which the chemical exerts its biological impact in 50% of the tested planktonic organisms. Therefore, for a potential non-toxic and environmentally safe antifouling agent, the LC50/EC50 ratio must be >15, emphasizing the need for relatively low EC50 values during experimentation [19]. These antifouling agents belong to the chemical classes of terpenoids, phenolics, steroids, polyketides, furanone, alkaloids, peptides, and lactones [20]. 

Following a summary of the types of fouling and the strategies to combat it, this review will emphasize the most recent antifouling agents that have been recovered from marine actinomycetes species along with a detailed description of their chemical compositions, classes, and biological activities. It will also feature a few prospects for the optimized production of natural antifouling agents with reliable biological activities, which can later be implemented for scaled-up industrial production of natural antifouling agents. 

## 2. Fouling: The Root Causes and Progression

Fouling is a term used to describe the deposition of undesirable organic or inorganic materials on external surfaces. There are two major types of fouling: non-biological and biological fouling (biofouling). Non-biological (also known as inorganic fouling) includes the accumulation of corrosions, oils, salt crystals, and ice on submerged surfaces. Biofouling is the undesirable deposition of organic elements secreted by micro- or macro-organisms (biofilm, EPS, etc.) over submerged surfaces [7,21,22]. As the habitual conditions and causative microorganisms vary, medical, maritime, and industrial fouling forms are very different from one another. While maritime and industrial biofouling are combinations of biofilm and macro- and inorganic fouling, medical biofouling is primarily made up of biofilm buildup [23]. 

Medical fouling may damage indwelling prosthetics, such as fasteners, prosthetic valves, bone plates, dental and orthopedic implants, pacemakers, long-drug-delivery devices, or short-term temporary medical devices, such as catheters, biosensors, ophthalmic lenses, drug-delivery devices, ventilation tubes [24]. *Enterococcus faecalis*, *Staphylococcus epidermidis*, *Staphylococcus aureus*, *Escherichia coli*, *Klebsiella pneumoniae*, *Pseudomonas aeruginosa*, *Proteus mirabilis*, and *Streptococcus viridans* are among the organisms that cause infectious biofilm deposition and medical fouling of catheters, tracheal tubes, and ventilators [25,26]. Medical fouling represents a significant burden on healthcare systems where >900,000 cases of UTIs in the US are catheter-related infections, which account for 40% of nosocomial infections [27]. Further, bloodstream infections due to fouled catheters are associated with 12–25% mortality and a high economic cost of $25,000 per case [28]. It is also reported that medical biofouling accounts for more than 45% of hospital-acquired infections, with 9% of every 9000 patients developing ventilator-associated pneumonia [29].

On the other hand, maritime fouling, which affects ships, sonar devices, pipelines, pillars, offshore infrastructures, oil installations and platforms, undersea cables, etc., is the most prevalent type of environmental fouling [23]. It might either be macro- or microfouling as described in Table S1 where macrofouling involves the attachment of bigger offending animals, such as barnacles, mussels, polychaete worms, and bryozoans, and microfouling involves the creation of biofilms by acid-producing bacteria [30,31,32,33,34,35,36,37].

**Figure 1:**
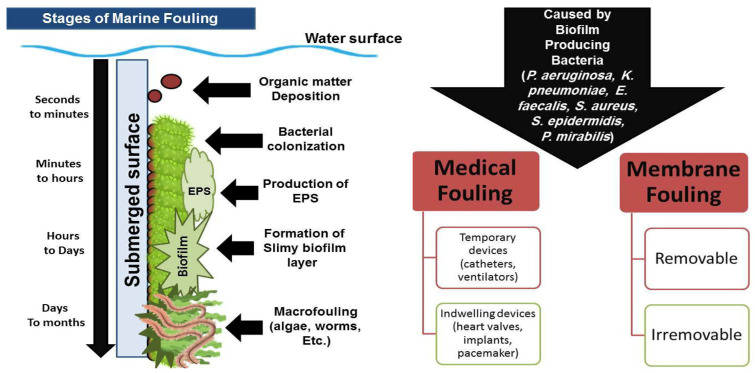
Stages and different forms of fouling.

After a few seconds from the initial exposure of the submerged surface and the aquatic habitat affluent in nutrients and microorganisms, the multistage process of marine biofouling takes place (Figure 1) [38]. Bacteria initially attach themselves to solid surfaces, colonize, and start secreting extracellular polymeric substances (EPSs). Electrostatic and Van der Walls interactions play a substantial role in the early phases of fouling when bacteria cling to the exposed surfaces. More complex communities, such as different multicellular species, microalgae, detritus, sediments, etc., are drawn to the damaged surface following the bacterial colonization and development of slimy biofilms. The macrofoulers, including barnacles, mussels, and macroalgae, are involved in the ultimate phases of fouling. Environmental factors, like pH, salinity, turbulent flow, temperature, and formation, have a significant impact on the progression of this process [38,39,40].

In industrial fouling, the shear forces might reduce the likelihood of biofilm deposition on industrial membranes. Although the active sludge produced by membrane filtrations may have strong shear pressures that may nevertheless have higher bacterial populations than in a marine environment, promoting the development of slimy biofilms and the initiation of membrane fouling [41]. Additionally, the constant hydrodynamic cross-flow across the membrane surface results in superior adhesion and improved quorum sensing (QS), increasing the rate of bacterial nourishment and improving aeration for the biofilm layer [42]. Membranes that are fouled can either be physically removed (removable) or cleaned up, or they cannot be removed and need chemical cleaning. Unfortunately, biofouling increases expenses and security risks in both marine and industrial environments [16]. 

## 3. Synthetic Antifouling Strategies and Toxicities Associated with Conventional Antifouling Coatings 

Various tactics are used to combat maritime, medical, and industrial fouling. The numerous surface factors that significantly affect biofouling include the surface’s wettability, texture, contours, and colors. Hydrophilic surfaces have high wettability and low surface energy, while hydrophobic surfaces have low wettability and high surface energy. Unexpectedly, surfaces with low adhesion and high hydrophobicity enhance contaminant clearance and self-cleaning. Surfaces with super hydrophilic textures can also show less protein adsorption and bioadhesive characteristics [23]. Additionally, matrix-assisted pulsed laser evaporation treatment of biomaterial surfaces was linked to a reduction in bacterial adhesion [23,43]. Synthetic self-cleaning hydrophobic paints, hydrophobic, nitrofurazone-containing polymer coats, and silver-based hydrogels are among the methods used to counteract various types of fouling. Additionally, reduced protein adsorption and bioadhesion are seen with super hydrophilic PEG coatings [23,43].

Sufficient antifouling coatings are also applied to affected surfaces while considering the stability of the hydration layer in the case of superhydrophilic layers, the rate of coating degradation, and the rate of antifouling agent release from the coating layer [3]. These coatings may have synthetic or natural origins. Antifouling coating materials have a variety of modes of action; some reduce macrofouling by inducing algal cellular Ca^2+^ efflux, which stops development and induces cellular arrest [44]. Others react by going through biological degradation after being exposed to specific ions, such as Na^+^, Cl^−^, which enable periodical self-renewal of polymeric surfaces [45]. Additionally, some coatings are switchable polymers that undergo reversible phase transitions with their surroundings, creating unfavorable conditions for bacterial attachment and growth [43]. 

TBT and its derivatives have been used in antifouling coats for commercial ships and hulls for the past decades. It belongs to the class of organic compounds named the trisubstituted organotin compounds (OTCs) and has been used as an antifouling agent along with the other organotin derivatives (monobutyltin (MBT), monophenyltin (MPT), and azocyclotin (ACT) [46,47]. TBT and organotin derivatives are very toxic to several aquatic species [48]. TBT acetate has been lethal to *Crassostrea gigas* oyster larvae at (50 ngL^−1^) with a no-observed-effect level conc. (i.e., NOEL) of 20 ngL^−1^, which denotes the minor difference between both concentrations and the toxicity of TBT [48]. Further, TBT leakage into bulk water has been associated with structural anomalies in oyster shells, including wafer-like chambering, hypersecretion of interlamellar jelly, and calcification [49]. It is also toxic to mollusks with a NOEL score < 1 ng/L, which placed some heavily exposed marine activities in jeopardy of continuity [49].

When compared to samples taken in 1986, the content of TBT in oysters (*Crassostrea gigas*) and mussels (*Mytilus edulis*) in 1989 was reduced by 25 to 33% after the UK passed legislation to regulate and control the sale of TBT. In the years that followed, oysters with regular shells grew normally with acceptable meat quality [50]. Additionally, organotin chemicals may disrupt the normal interaction between sex hormones and their receptors and impair steroid receptor signaling according to molecular docking studies [46,47]. 

Antifouling paints have also incorporated both irgarol 1051 and diuron for their algicidal activities. It has been demonstrated that irgarol 1051 and diuron at concentrations above 0.5 μg/L reduce the seagrass *Zostera marina*’s photosynthetic abilities, which is associated with a prominent reduction in their growth [51]. Depending on the quality of the water and environmental parameters, like temperature, salinity, light, current, etc., the effects of copper-based antifouling chemicals are only transient, lasting between 5 and 12 months [11]. They are best suited for seawater and extremely harmful to freshwater and riverine algae and mollusks. On the other hand, zinc pyrithione found in several antifouling coatings has been found to be exceedingly toxic to aquatic plants and animals despite the fact that it is assumed to be environmentally safe due to its propensity to photo-degrade into less toxic chemicals [11,52]. Sea-Nine has also been utilized as an antifouling coating, yet it was toxic to various aquatic species [11,53]. 

Since then, numerous studies have been dedicated to finding the optimal antifouling coatings that are biocidal to fouling creatures yet capable of maintaining normal ecological balance (safe and non-toxic to aquatic creatures). An ideal antifouling agent must develop sufficient antifouling activity while imposing the least amount of hazardous effect on the maritime organisms that inhabit the surrounding environments of submerged surfaces. Different types of recently developed antifouling coatings that are utilized to prevent medical fouling [54,55,56,57,58,59,60,61,62,63,64,65,66,67,68,69] and maritime/environmental fouling [70,71,72,73,74,75,76,77,78,79,80,81,82] are listed in Table S2. 

## 4. Natural Antifouling Agents (AFs) as an Alternative to Synthetic Antifouling Coatings

Natural AFs are alternatives to synthetic antifouling coatings that are environmentally benign with acceptable compatibilities. Effective fouling prevention without long-lasting negative environmental effects is the key characteristic of an excellent natural antifouling agent [83]. One of the primary sources of production for such chemicals is marine microorganisms. AFs function as inhibitors of metabolic signaling pathways, neurotransmitter disruptors, or anti-bioadhesives that eventually block larval settlement (anti-macrofoulers). While neurotransmitter disrupters cause invertebrate larvae to resist settling, anti-bioadhesive AFs work by altering the proteinaceous surface to remove the surface-level inductive hints [19]. When a natural AF’s fatal dose (LC50) to minimal concentration inhibiting settling (EC50) ratio (LC50/EC50) is more than 15, it is deemed non-toxic [19,83]. Natural antifouling agents belong to different chemical classes; they may be terpenoids, steroids, fatty acids, alkaloids, benzenoids, proteins, or quorum-sensing inhibitors. Each of those has a unique method of operation [83]. 

The terpenoids (terpenes) extracted from the red alga *Laurencia rigida* prevent the larvae of *Amphibalanus amphitrite* (also known as *Balnus Amphitrite*) and *Bugula neretina* from forming settlements [84,85]. *L. rigida* extracts are rich in terpenoids, elatol, and deschloroelatol; anti-settlement activity against the barnacle larvae was tested using cyprids of *Amphibalanus amphitrite* (also known as *B. amphitirite*) against three synthetic antifouling agents, irgarol 1051, Sea-Nine 211, and nopcocide N-96. Both terpenoids inhibited the attachment of *B. amphitirite* cypris larvae at a conc. of 10 ng cm^−2^ with etalol inhibiting 100% of larval settlement and deschloroelatol exerting 90% inhibition. The control synthetic antifouling agents were, however, less effective than elatol and deschloroelatol. Irgarol 1051 was the least active compound at 10 ng cm^−2^. It is interesting to highlight the eminent lethality of both elatol and deschloroelatol to *B. amphitrite nauplii* (early larval stages), incurring 100% mortality at 100 ng cm^−2^ and almost 90% and 50–60% mortality at 10 and 1 ng cm^−2^, respectively. Lethality to *B. amphitrite nauplii* has been abolished upon experimenting with either nopcocide N-96 or Sea-Nine 211 at 10 ng cm^−2^. Both terpenoids acquire anti-adhesive potentialities since they successfully hindered the bryozoan *Bugula neretina* larvae from settlement formation at 10 μg cm^−2^ [84]. 

Halogenated terpene derivatives are also useful natural AFs in the prevention of larval adhesion. *Sargassum tenerrimum* phlorotannins hinder the metamorphosis of *Hydroides elegans* [86]. *S. tenerrimum* extract is opulent with phlorotanins, phloroglucinol, and tannic acid. The compounds elucidate variable anti-adhesive potentialities against *Hydroides elegans* larva settlement. Phlorotanins attain high safety profiles since the reported LC50 score was 27 times higher than their EC50 score, which were 13.984 ppm and 0.526 ppm, respectively. This gives an LC50/EC50 score of 26 for *S. tenerrimum* phlorotanins. Phloroglucinol reports an EC50 score of 5.231 ppm and an LC50 score of 206.823 ppm (LC50/EC50 ratio of 38). Further, tannic acid has the highest activity and toxicity with EC50 and LC50 scores of 4.032 × 10^−3^ and 4.090 ppm, respectively. The three compounds, phlorotanin, pholoroglucinol, and tannic acid, have bactericidal effects against eight bacterial species that regulated larval settling, with tannic acid elucidating the best inhibiting capabilities at 1 mg disc^−1^ [86]. 

In similar manners, the brown alga *B. bifurcate* extract opulent with phlorotannins and other terpene derivatives demonstrates antibacterial vigor against both biofilm-forming bacteria *Cobetia marina* and *Pseudoalteromonas haloplanktis* with an MIC score < 50 μg/mL. Additionally, the extract has an anti-larval settlement effect against *Amphibalanus amphitrite* cypris larvae with an EC50 score below 15 μg/mL and a naupliar toxicity LC50 score of 38.3 μg/mL [87]. 

At non-toxic doses, the gorgonian *Junceella juncea* diterpene extract exhibits potent antifouling activity against *A. amphitrite* larval colonization [88]. The *J. juncea* extract rich with briarane diterpene, including juncin ZII, attained moderate insecticidal and antifeedant activity against *Spodoptera litura* second-instar larvae when compared to synthetic material, azadirachtin. When tested against *Balanus amphitrite* settlement, juncin ZII exerted significant anti-settlement effects with an EC50 score of 0.004 μg/mL [88]. The brown seaweed *Canistrocarpus cervicornis*’ dolastane, seco-dolastane diterpene, and isolinearol affluent extract hinder *Perna perna* mussel settlement [89]. The seco-dolastane successfully repressed 82% of mussel *P. perna* from settlement, while dolstane halted the byssal attachment by 60% (*p* < 0.05) [89]. The bromosphaerol, sphaerococcenol A, and bromotetrasphaerol derived from *Sphaerococcus coronopifolius* are effective anti-macrofouling agents against *Balanus amphitrite* larvae colonization [90]. Both Bromosphaerol and bromohydrin derivatives attain impressive safety profiles with an LC50/EC50 ratio of 434 (EC50 score of 0.23 mg/L and LC50 score > 100 mg/L) [91].

The *Streptomyces tumemacerans* albofungin is a potent antifouling agent with an equivalent efficacy to butenolide against *Amphibalanus amphitrite* larvae and an EC50 score of 1.6 μg/mL. It also acquires acceptable safety profiles with an LC50/EC50 ratio > 100 even when used at high concentrations (up to 40 μg/mL) [92]. The anti-adhesive characteristics of the previously mentioned natural antifouling agents may be assigned to the inhibition of the phenoloxidase and tyrosinase enzymes that regulate the crosslinking and creation of the adhesive plaques needed to anchor the mussels’ byssal and substrata. This was observed when arctic marine sponge *Stryphnus fortis* bromotyrosine-rich extract blocked blue mussel phenoloxidase and hindered their settlement [93]. 

The first known antifouling benzenoid, 3-chloro-2,5-dihydroxybenzyl alcohol, retrieved from *Ampelomyces* sp. UST040128, is known for its anti-larval settlement effect against both *Balanus amphitrite* cyprids and *Hydroides elegans* larvae. Its EC50 score for *B. amphitrite* ranged from 3.19 μg/mL to 3.81 μg/mL, and the LC50 score was 266.8 μg/mL. Upon testing on *Hydroides elegans*, the observed effect was dose-dependent, and the EC50 score was 0.67 μg/mL to 0.78 μg/mL, and the LC50 value was 2.64 μg/mL [94]. 

Amibromdole isolated from *Sarcophyton* sp. fungus is a halogenated benzenoid with powerful antifouling activity against *Balnus amphitrite.* Similarly, pestalachlorides E and F secreted by the fungal strain *Pestalotiopsis* ZJ-2009-7-6 elucidates strong antifouling activity against *B. amphitrite* larval settlement [18]. Both compounds have EC50 scores ranging from 1.65 to 0.55 μg mL^−1^ against the barnacle *Balanus amphitrite*’s larval settlement with an LC50/EC50 score > 15, suggesting their safety and efficacy [95]. 

Dihydroquinolin-2-one-containing alkaloids isolated from *Scopulariopsis* sp. fungal extracts are known for their eminent anti-macrofouling vigor against *Balanus amphitrite* larval colonization with acceptable safety and therapeutic profiles [96]. The dihydroquinolin-2-ones have average EC50 scores of ~25–50 μg/mL and LC50 scores of ~3.79–7.85 μM when tested against the brine shrimp *A. salina* [96]. They are also known for their bactericidal effects against the fouling bacterial species *S. aureus*, *B. cereus*, *V. parahaemolyticus*, *N. brasiliensis*, and *P. putida*, with MIC scores of 0.78, 1.56, 6.25, 0.78, and 1.56 μM, respectively [96]. Further, the indole alkaloid including the spiro-anthronopyranoid-diketopiperazin purified from gorgonian *Eurotium* sp. fungus attains anti-macrofouling activity against *B. amphitrite* larvae [97]. However, the study denoted that the addition of anthronopyranoid moiety to diketopiperazine is associated with a minor reduction in antifouling vigor, as a slight elevation in the EC50 score from 15–17 μg/mL to >50 μg/mL was observed. Unexpectedly, the teratogenicity of the indole alkaloid in the zebrafish model was lowered following the introduction of an anthronopyranoid moiety [97]. 

The N-methyltetrahydroellipticine and furoquinoline alkaloids, kokusaginine and flindersiamine, purified from Atlantic yellow guatambu’ *Aspidosperma australe* and white guatambu’ *Balfourodendron riedelianum* trees elucidate eminent anti-macrofouling potential against the *Mytilus edulis* platensis mussel [98]. *A. australe* bark extract was opulent with pyridocarbazole olivacine, indole alkaloids uleine, and N-methyltetrahydroellipticine, where N-methyltetrahydroellipticine yielded the best anti-adhesive macrofouling activity with an EC50 value of 1.56 nmol cm^−2^ against *Mytilus edulis platensis* mussels. Second to N-methyltetrahydroellipticine in its anti-macrofouling vigor is the kokusaginine retrieved from *B. riedelianum* bark extract that scored an EC50 value of 3.86 nmol cm^−2^. The others, flindersiamine, olivacine, and uleine, recorded EC50 scores of 5.56, 7.59, and 9.95 nmol cm^−2^, respectively. This study denotes the advantageous usage of *M. edulis platensis* mussels in an anti-macrofouling bioassay exhibiting better reproducibility for small laboratory scale testing. The N-methyltetrahydroellipticine and kokusaginine coats are also efficient anti-macrofouling agents when tested in ocean water for 45 days. Both coats halted the adhesiveness and settlement of green and red alga, *Ulva intestinalis* and *Griffithsia* sp., respectively. They also prevented colonial species settlement (bryozoan *Bugula* sp., tunicate *Botryllus* sp.). The anti-macrofouling activity was assigned to the furan ring in kokusaginine, which is known for acquiring several biological activities. Furthermore, furoquinolone alkaloids can efficiently inhibit the acetylcholinesterase enzyme (AchE) and exert antifeedant and antibacterial activity [98].

Further, the inhibition of the AChE is seen among the territerm derivatives isolated from marine-derived *Aspergillus terreus* fungus. Suppression of the AChE blocks cholinergic signaling and neurotransmission, which in turn disrupt the barnacle cyprid settlement and induce a hermetic behavior [99]. Penilloid A, an indolyl diketopiperazine alkaloid, purified from *Penicillium* sp. and *Aspergillus sydowii* fungus demonstrates prominent anti-macro-antifouling activity against both *B. amphitrite* and *B. neritina* larvae and antibacterial activity against larval settlement, inducing bacterium *Micrococcus luteus* (MIC 200 μg/mL) [100]. 

The avoidance of quorum sensing is another effective strategy to deter marine fouling. Autoinducers (AIs) are used by bacteria to communicate in the early phases of fouling. When the concentration of AIs reaches a particular threshold, changes in bacterial genome expression take place, and the bacteria begin to secret extracellular polymeric substances and initiate biofilm formation. While Gram-positive bacteria utilize oligopeptides as autoinducers, Gram-negative bacteria communicate via N-acetylated homoserine lactones (AHLs) [101,102]. Similar-looking molecules to AHLs may prevent cell-to-cell contact and obstruct quorum sensing. The marine actinomycete *Streptomyces* sp. produces butenolides, an efficient anti-microfouling substance that effectively prevents the growth of biofilms and eliminates pre-existing slimy biofilms caused by *E. coli*, *P. aeruginosa*, and *MRSA* [103,104]. It is noted that the addition of a tert-butyloxycarbonyl (Boc) and 7-carbon alkyl side chain to the terminal amine of butenolides was associated with better antifoulant activities [105]. Table 1 shows some examples of natural AFs that can be used as antifouling coatings to prevent different forms of fouling.

## 5. Marine Actinomycetes as Sources of Natural Antifouling Agents

Actinomycetes, in particular, marine actinomyctes, are highly important industrial sources of secondary metabolites that include a wide range of antimicrobial, antibacterial, and antifouling agents. They belong to the Gram-positive bacterial order Actinomycetales and display a wide range of distinctive features, such as habitat, ideal pH, thermophilicity, and moisture tolerance. They interact with a wide range of aquatic animals, including invertebrates, like sponges, corals, and echinoderms, as well as vertebrates, like pufferfish corals, and a variety of invertebrates [130,131]. The evolution of secondary metabolic pathways may be influenced by these interactions, which may promote particular chemical ecologies. Although most strains have been identified from sediments, marine actinomycetes can coexist with other species and live in both planktonic and biofilm habitats [15].

Interestingly, marine actinomycetes secrete various antifouling agents that have proved efficacious and safe. These metabolites belong to various chemical classes, including peptides, polyketides, isoprenoids, sterols, and phenazines. *Streptomyces* sp. is known to produce terpenoids and steroids, fatty acids, and quorum-sensing inhibitor antifouling agents [109,130]. *Norcadiopsis* and *Rubrobacter* sp. are known producers of amino imidazoles and diketopiperazines alkaloids [132]. Antifouling terpenoids have been detected among the extracts of actinomycetes belonging to *Micromonosporaceae*, *Nocardiaceae*, and *Pseudonocardiaceae* [92,93]. Additionally, marine actinomycetes retrieved from marine algae have specified enzymatic activities that facilitate heavy metal sorption from the marine environment and attain flocculating activities. Along with producing 56 U/mL of amylase, *Streptomyces* sp. SNAJSM6 has outstanding bactericidal action against a number of MDR pathogenic bacteria, including *Micrococcus luteus*, *Enterobacter* sp., *Salmonella* sp., and *P. aeruginosa*. *Nocardiopsis* sp. GRG 3 extracts have prominent metal sorption abilities that successfully cleared 51.90% Hg, 74.7% Pb, 85.90% Cr, and 55.90% Cd [133]. The natural antifouling compounds produced by the families of marine actinomycetes, together with their molecular mechanics and toxicity profile, are listed below.

### 5.1. Antifouling Agents from the Streptomycetaceae Family

Several bioactive compounds that are helpful in various aspects (agriculture, biotechnology, etc.) are generated within microbes belonging to the *Streptomycetaceae* family. Streptomyces is the most investigated genus among *Streptomycetacea* members, and several of its species have been found to have insecticidal, larvicidal, pesticidal, acaricidal, antifouling, and nematocidal action [17]. *Streptomyces* sp. is non-motile, filamentous, Gram-positive bacteria with aerial hyphae that generate long spore chains (>50) [134]. Numerous antifouling substances are secreted by *Streptomyces* sp.; these include terpenes, alkaloids, and quorum-sensing inhibitors. 

#### 5.1.1. *Streptomyces* sp. Terpenoids

Oxycyclopentadien, also known as 1,3-cyclopentadien-1-ol, is a terpenoid produced by the bacterium *Streptomyces thermolineatus* VITKV6A that was identified from a rhizosphere soil sample. The substance demonstrated strong anti-microfouling action against the biofilm-forming bacteria *Kocuria rhizhophila*, *Psychrobacter alimentarius*, and *Psychrobacter celer*, each having MICs of 0.75, 0.75, and 0.5 μg/mL, respectively. Using the mollusk foot adherence assay at 1000 μg mL^−1^ also revealed anti-macrofouling activity against *Patella* sp. with acceptable cytotoxicity and an LC50 value of 173.72 μg mL^−1^ against *Artemia salina*. It is interesting to note that all *Patella* sp. functions, including spreading, contracting, and attaching its foot, were affected by the compound concentration. The fact that the organisms could survive even at the greatest tested levels proved the substance was not harmful. The result demonstrates the compound’s environmental friendliness while inhibiting mollusk foot adhesion. The single peak seen in the HPLC chromatogram with a mass spectrum molecular weight of 83.91 g mol^−1^ and the broadband within the FTIR spectrum at 3387 cm^−1^ along with the bands at 2941 cm^−1^, 2831 cm^−1^, 1435 cm^−1^, 1022 cm^−1^, 914 cm^−1^, and 729 cm^−1^ indicated the presence of hydroxyl, C-H, C-O, and C=C functional moieties. The 13C NMR investigations revealed the presence of five carbon atoms in the molecule, which is a shared property for all terpenoids. The carbon with the oxygen bond was discovered at 115.03 shift, the carbons with double bonds were discovered in the region within 38.71 to 51.90 (3 shifts), and the individual CH_2_ was discovered at 28.57 shift in the beginning [135,136].

Triterpene glycosides found among *Streptomyces fradiae* RMS-MSU extracts exhibit anti-microfouling vigor. The triterpene glycosides recorded the highest inhibition zone (19–21 mm) among the cultivated *E. coli*, *Pseudomonas sp1*, *H. aquamarina*, *Vibrio* sp., *A. hydrophila*, *C. freundii*, *S. sonaii*, and *S. fonticola* growths; that was followed by an inhibition zone of 18 mm among *M. morganii* and *S. liquefaciens* growths. When examined against *Enterobacter* sp., *Micrococcus* sp., *Salmonella* sp., and *V. parahaemolyticus*, RMS-MSU triterpene glycoside extracts elucidated minor anti-microfouling vigor, scoring an inhibition zone of 10–12 mm. According to these findings, RMS-MSU crude extract possesses a potential bacteriostatic vigor toward Gram-negative bacterial species with the lowest scored MIC of 25 μg mL^−1^. The MIC scores increased gradually to 50–200 μg mL^−1^ when the antibacterial vigor of the RMS-MSU crude extract was examined on *A. hydrophila*, *M. morganii*, *C. freundii*, *P. pudita*, *H. aquamarina*, *S. liquefaciens*, *S. mercescens*, and *Enterobacter* sp. Interestingly, the RMS-MSU triterpene glycosides extract successfully exerted anti-microalgal effects at 100–200 µg mL^−1^ against *Chlorella* sp., *Nannochloropsis* sp., and *Dunaliella* sp. and 200 µg mL^−1^ for *Chaetoceros* sp. and *Tetraselmis* sp. The triterpene glycoside extracts also showed good anti-crustacean efficacy against *A. salina* and exhibited an LC50 of 718.79 μg mL^−1^ with 50% mussel mortality. The therapeutic ratio (LC50/EC50) of 9.33 indicated that the extract acquires a benign nature. When transferred to fresh seawater, the limpet *Patella vulgata* demonstrated 6.66% fouling and 92.96% recovery in the mollusk foot adherence experiment [137].

The organic extracts of *Streptomyces aculeolatus* PTM-420 opulent with napyradiomycins (SF2415B3, 4-dehydro-4a-dechloro-napyradiomycin SF2415B3, A80915A, A80915C, A80915A, 18-hydroxynapyradiomycin A1, 16-oxonapyradiomycin A2, 4-dehydro-4a-dechloro-16-oxonapyradiomycin A2, 4-dehydro-4a-dechloro-napyradiomycin B3) exhibits *C. marina* antibacterial activity. The overall in silico toxicity profiles of napyradiomycin pointed to a minimal bioaccumulation factor, the absence of mutagenicity, and a toxicity comparable to that of commercially available medicines and antifouling biocides. To assess the EC50 of napyradiomycins, in vivo experiments were conducted using *Mytilus galloprovincialis* mussel stick larvae and sequential concentrations of napyradiomycins. Napyradiomycins showed an anti-settlement effect without viability impairment (EC50 < 5 µg/mL and LC50/EC50 > 15) [138]. 

The *Streptomyces kebangsaanensis* WS-68302 strain also secretes two novel napyradiomycins derivatives, napyradiomycin A4 and A80915 H, with the A4 derivative exerting potent antiviral activity against *Pseudorabies* virus at 2.056 μM and a therapeutic ratio of 14.98 [139]. As previously reported, the meroterpenoids, naphterpin, nitropyrrolin, and marinophenazine were already retrieved from marine *Streptomyces* sp. CNQ-509. However, the CNQ-509 strain has the ability to produce two novel naphterpin derivatives that comprise the known terpenoid debromomarinone along with naphthoquinone and geranyl moieties [140]. 

The novel furaquinocins K and L belonging to naphthoquinone-based meroterpenoids are opulent among extracts of the marine *Streptomyces* sp. Je 1–369 strain. The presence of an acetylhydrazone fragment within the polyketide naphthoquinone skeleton of furaquinocins L differentiates them from the originally recovered furaquinocins. Both furaquinocins K and L elucidate Gram-positive bactericidal effects against *B. subtilis* DSM 10 and *S. aureus* with MIC scores of 64 μg/mL and 2 μg/mL, respectively. The furaquinocins K are also lethal to hepatocellular carcinoma (HepG2) cells with an IC50 score of 12.6 μg/mL [141]. 

The merochlorins G–J, the chlorinated tetrahydroxynaphthalene (THN)-derived meroterpenoids, are identified among the cultures of marine *Streptomyces* sp. CNH-189. They are synthesized by connecting the THN with a C15 isoprene unit through Baeyer-Villiger-style oxidation, a Paterno-Büchi-type 2 + 2 cycloaddition, and a pinacol-type contraction. There are two classes of tetrahydroxynaphthalene (THN)-derived meroterpenoids: Class I, which includes neomarinone, merochlorin A-B, and Class II merochlorins G–J. These compounds (merochlorins G–J) harness an isoprene unit at C3 of their THN skeleton. Merochlorins G, H, and I are the chlorinated versions of merochlorin D at C-18, whereas merochlorin J is the cyclized version with an additional amine at C19 and a tetrahydrofuran ring moiety. Merochlorins attain antibacterial activity against *Bacillus subtilis*, *Kocuria rhizophila*, and *Staphylococcus aureus* with MIC scores ranging from 1–2 μg/mL. Only merochlorin G has marginal antibacterial activity against the tested microorganisms with MIC scores ranging from 16–32 μg/mL [142].

Guanahanolide A is a new meroterpenoid that has been isolated from the fermentation extract of *Streptomyces* sp. RKBHB7. This compound harnesses a dihydronaphthalenone moiety and a perhydrophenalene-like sesterterpene carbon skeleton. Sesterterpene is not a common moiety among secondary bacterial metabolites. Guanahanolide A resembles halimane and labdane diterpenoids, such as actinoranone. Actinoranone is a dihydronaphthalenone polyketide linked to a bicyclic diterpenoid secreted by *Streptomyces* sp. [143]. Guanahanolide A lacks any antibacterial activity against *MRSA*, *Staphylococcus warneri*, *Pseudomonas aeruginosa*, *Proteus vulgaris*, or *Candida albicans.* It is only cytotoxic to various cancerous cell lines [144]. Table 2 summarizes the chemical formula, structure, and mechanisms of *Streptomyces* sp. terpenoids.

#### 5.1.2. *Streptomyces* sp. Alkaloids 

The piperidine alkaloids strepchazolins A, B purified from the marine actinomycete *Streptomyces chartreusis* NA02069 are purportedly antibacterial agents with mild in vitro inhibitory activity against acetylcholinesterase (AChE) and an IC50 score of 50.6 μM. The strepchazolins harness both cyclopentene and tetrahydropyridine moieties. Strepchazolins B is the diastereoisomer of strepchazolins A. The streptchazolin A exhibits the uppermost activity, recording an MIC value of 64.0 μM against *Bacillus subtilis*, and acquires modest acetylcholinesterase (AChE) inhibitory activity [155]. As previously mentioned, tyrosinase (Tyr) and AChE are enzymes linked to adhesive mechanisms during biofouling species colonization. Inhibition of either is associated with antifouling activity [156,157]. 

Likewise, *Streptomyces* sp. ZZ741 secretes glutarimide alkaloids resembling the streptoglutarimides A–J, which harness additional oxide groups linked to their ring structure but share the same piperidine skeleton. The ^1^H NMR spectra revealed the existence of fifteen carbons, including three carbonyls with a wavelength of 1691 cm^−1^ in the FTIR spectrum, two olefinic carbons, one non-protonated carbon connected to oxygen, one oxymethylene, two methines, four methylenes, and two methyls. The chemical formula of streptoglutarimides calls for six degrees of unsaturation with three carbonyls and two rings where the first is the glutarimide moiety and the second is the tetrahydrofuran derivative. The streptoglutarimides are powerful antibacterial and antifungal agents with MIC scores of 9−11 μg/mL against MRSA, 8−12 μg/mL against *Escherichia coli*, and 8−20 μg/mL against *Candida albicans* [158]. 

The *Streptomyces anulatus* S71 strain identified from a marine sponge *Aplysina aerophoba* organic extracts are opulent with glutarimide-derived compounds and one indole alkaloid. These include 3-[2-[2-hydroxy-3-methylphenyl-5-(hydroxymethyl)]-2-oxoethyl] glutarimide, 3-[2-(2-hyroxy3,5- dimethylphenyl)-2-oxoethyl] glutarimide, also known as actiphenol, 3-hydroxy-3-[2-(2-hydroxy-3,5-dimethylphenyl)-2-oxoethyl] glutarimide, and 3-[2-[2-hydroxy-3 (hydroxymethyl)-5-methylphenyl]-2-oxoethyl] glutarimide, along with a known indole, alkaloid 3-(hydroxyacetyl) indole [159]. The capacity of the glutarimide alkaloid to inhibit AChE suggests an anti-macrofouling capability [160]. The polyketide streptimidone, which contains glutarimide, is released by *Streptomyces* sp. W3002 and exerts mild cytotoxic action against HeLa, Hep3B, and HL-60 [161].

The tricyclic quinolizidomycins A and B purified from *Streptomyces* sp. KIB-1714 is a potential anti-macrofouling agent for its AChE inhibitory activity [162]. In the FTIR spectra, the quinazolinone alkaloids exhibit significant absorption bands at 3406 cm^−1^ and 1675 cm^−1^ for NH and conjugated carbonyl groups, respectively. According to H 1 and COSY spectroscopic results, they also contain a distinctive quinazoline-4-one component. Additionally, they are known for their lethality on normal cell lines (Vero cells) with an IC50 score of 3.30 μg/mL [163]. 

*Streptomyces* sp. HZP-2216E produces indolizinium alkaloids and zwitterion streptopertusacin A that acquire modest antibacterial vigor against Gram +ve MRSA [164]. Its zwitterionic nature may suggest its antifouling potentiality. The cyclizidine-type alkaloids are another indole-based alkaloid purified from marine *Streptomyces* sp. HNA39. According to the 1D-NMR spectra, the cyclizidine alkaloids contain the characteristic monosubstituted cyclopropyl ring, two methyl groups, three olefinics, four methylenes, three methines, one oxygenated carbon, and non-protonated carbons. Double bonds and hydroxyl functionalities are also indicated by the FTIR spectrum at 1683 cm^−1^ and 3421 cm^−1^, respectively. These substances have cytotoxic effects and ROCK2-protein kinase-inhibiting activity [165,166]. 

Geranylpyrrol A derivatized from the extracts of *Streptomyces* sp. CHQ-64 stain harbors a pyrrol-based skeleton with seven non-protonated carbons (two carbonyls and five aromatic/olefinic). The chemical shifts from C-1 to C-9 during the 1H and 13C NMR investigations confirm the presence of a geranyl chain. This compound is lethal to different cell lines [167]. 

Like geranylpyrrol A, the anandins A and B, prevalent among *Streptomyces anandii* extracts, comprise pyrrole rings and elucidate temperate antibacterial performance [168,169]. The glycosylated piericidins glucopiericidinol A3 and 7-demethyl-glucopiericidin A were found among *Streptomyces* sp. KIB-H1083 cultures and contain pyridine rings in their structures. Piercidin A exerts the uppermost antibacterial activity against *Xanthomonas oryzae pv. oryzicola* and *Penicillium decumbens* [170]. Malaymycin is a brand-new tetrahydroquinoline alkaloid that contains cyclopentenone. McCreamycin E is a geldanamycin analog with a rare cyclopentenone moiety and a C2-symmetric macrodiolide that was opulent among the extracts of *Streptomyces malaysiensis* SCSIO 41397. These compounds were extremely cytotoxic [171]. Furthermore, the halogenated carbazole core compounds thiocarbazomycins A–B, chlocarbazomycin E, brocarbazomycin A, and chlocarbazomycins A–C are prevalent among the extracts of *Streptomyces diacarni* SCSIO 64983 [172]. Aranciamycin K and isotirandamycin B, pyrrolidines-based alkaloids, were isolated from a marine-derived *Streptomyces* sp. SCSIO 41399 along with the previously reported tirandamycin derivatives. Both compounds exert prominent bacteriostatic effects against *Streptococcus agalactiae* with an average MIC value of 5.9 μM [173]. 

The first antimycin-type antibiotics with a branched side chain were urachimycins A and B. *Streptomyces* sp. *THS-55* provides novel antimycins (antimycin A2c) that prevent *Candida albicans* from differentiating morphologically. Antimycin A2c also acquires mitochondrial degrading abilities that successfully destruct HPV E6/E7 oncoproteins, suggesting its potential usage for the treatment of cervical carcinomas [174]. *Streptomyces* sp. *182SMLY* secretes new phenazines alkaloids called streptophenazines A-H. The streptophenazines elucidate a broad-spectrum antibacterial action against both Gram-positive and -negative bacteria. With an MIC value of 4.2 μg/mL, streptophenazine B inhibited methicillin-resistant *Staphylococcus aureus* growth [175]. The frigocyclinone retrieved from the extracts of sponge-related *Streptomyces* sp. *M7_15* has eminent bactericidal activity with an EC50 score of 0.73 μM on several Gram-positive bacterial species [176]. Table 3 summarizes the chemical formula, structure, and mechanisms of *Streptomyces* sp. alkaloids.

#### 5.1.3. *Streptomyces* sp. Antibiofilm and Quorum Sensing Inhibitors

It is interesting to note that different bacteria’s ability to produce biofilms was greatly reduced by the wasted culture media of *Streptomyces parvulus* strain HY026. The active ingredient F1-4, also known as actinomycin D, which forms a considerable amount of the culture spent, considerably reduces the amount of violacein produced by *Chromobacterium violaceum* while having no inhibitory effect on bacterial growth at a dosage of 12.5 mg/mL. It also prevents prodigiosin production by *Serratia proteamaculans* with a 13.5 mm pigment inhibition zone at a concentration of 25 mg per disc. Additionally, waste media containing actinomycin D prohibit the biofilm formation by *Pseudomonas aeruginosa* PAO1, *Staphylococcus aureus*, *Micrococcus luteus*, and *Ruegeria* sp. in a dose-dependent manner [183]. When used against *P. aeruginosa PAO1*, actinomycin D significantly minifies the motility and down-regulates the expression of other virulence determinants, including pyocyanin, protease, rhamnolipid, and siderophores. It also attains prominent QSI ability, as it inhibited the production of N-(3-oxododecanoyl)-L-homoserine lactone and N-butanoyl-L-homoserine lactone [184]. *Streptomyces xanthocidicus* KPP01532 extracts opulent with piericidin A and glucopiericidin A elucidate prominent QSI abilities against the phytopathogen *Erwinia carotovora *subsp. *Atroseptica* [185].

Albofungin A, chrestoxanthone A, and chloroalbofungin isolated from *Streptomyces chrestomyceticus* BCC 24770 have eminent antibiofilm and QSI activities against the fouling bacteria *Staphylococcus aureus*, *Micrococcus* sp., *Staphylococcus* sp. with MIC scores of 0.03 to 0.5 μg mL^–1^, 0.06 to 0.5 μg mL^–1^, and 1.25 ng mL^–1^ to 0.2 μg mL^–1^, respectively. These compounds are able to suppress the Gram –ve *Pseudomonas pachastrellae* biofilm formation ability *with* an MBIC_90_ value ranging from 0.02 to 0.50 μg mL^–1^. Not only did the albofungin acquire antibiofilm activity, but it also attained anti-macrofouling vigor against the larval colonization of *Amphibalanus amphitrite* and *Bugula neritina* with an EC_50_ value of 2.5 μg [186]. 

**Figure 2:**
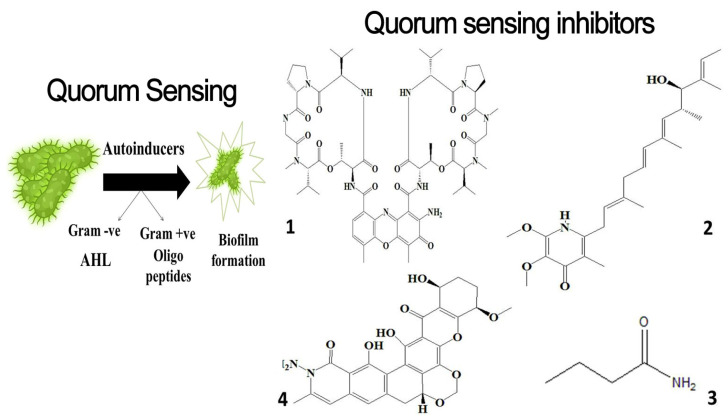
Quorum sensing inhibitors retrieved from *Streptomyces* sp. 1: actinomycin D; 2: piericidin A; 3: butanamide; 4: albofungin.

Similarly, cultures of *Streptomyces* sp. PNM-9 opulent with 2-methyl-N-(2′-phenylethyl)-butanamide and 3-methyl-N-(2′-phenylethyl)-butanamide suppressed the growth of phytopathogenic bacteria *Burkholderia glumae* with MIC scores of 2.43 mM and 1.21 mM, respectively. This suggests the possible usage of these substances to combat phytopathogenic microorganisms [187]. Cultures of halophile marine *Streptomyces* sp. deter biofilm formation by *Proteus mirabilis* and *Serratia marcescens* strains through the inhibition of quorum-sensing-regulated prodigiosin biosynthesis [188]. Furthermore, *Streptomyces* sp. cultures purified from the guts of Indian mackerels *Rastrelliger kanagurta*, *Panna croaker*, and *Panna microdon* elucidate antibacterial vigor towards *Staphylococcus aureus MTCC96*, *Escherichia coli MTCC739*, *Salmonella enterica*, *Candida albicans* and QSI activity against *Chromobacterium violaceum* and *Serratia marcescens* [189]. It is also noted that the biosurfactants retrieved from marine *Streptomyces althioticus* RG3 and *Streptomyces rimosus* NRRL 2455 exhibited good activities against *Pseudomonas aeruginosa*, *Klebsiella pneumoniae*, *Escherichia coli*, methicillin-sensitive *Staphylococcus aureus* (MSSA), and methicillin-resistant *Staphylococcus aureus* (MRSA) [190]. Figure 2 demonstrates the chemical structures of the quorum sensing inhibitor retrieved with *Streptomyces* sp.

### 5.2. Antifouling Agents from Micromonosporaceae, Nocardiaceae, and Pseudonocardiaceae Families

The actinomycetes *Micromonosporaceae* family includes the *Micromonospora* genus that develops highly branched substrate hyphae but infrequently shows sparse aerial hyphae [191]. The aerobic actinomycetes make up the *Nocardiaceae* family, which is distinguished by filamentous growth and genuine branching. *Nocardia* sp. is a catalase- and urease-positive organism that can grow on a variety of media, including basic blood agar, a Löwenstein–Jensen medium, and Sabouraud dextrose agar [192]. Finally, the solitary member of the suborder *Pseudonocardineae* is the family of bacteria known as *Pseudonocardiaceae*, and they include several genera, *Actinocrispum*, *Haloactinomyces*, and *Allosaccharopolyspora* [193]. These families have recently garnered a lot of attention as a prospective source of new, physiologically significant chemicals.

#### 5.2.1. Antifouling Agents from *Micromonospora* sp.

Marine *Micromonospora* sp. are rich sources of natural antimicrobial and antiparasitic agents. The dibenzodiazepine alkaloid diazepinomicin is affluent among the cultures of marine *Micromonospora* strain DPJ12. This alkaloid comprises a dibenzodiazepine moiety and a farnesyl residue. Diazepinomicin exerted anti-protease enzymatic activity at an IC50 score of 70–90 µM along with its acceptable antimicrobial activity and trypomastigote antiparasitic performance towards *Trypanosoma brucei* at an IC50 score of 13.5 μM [194,195]. The macrolides megalomicins A, B, and C, which are released by *Micromonospora* sp., have notable antiviral, antiparastic, and antibacterial activities, which resemble erythromycin C in their metabolic processes. Megalomicin A’s antiparasitic effect is related to the blockage of vesicular transport between the medial- and trans-Golgi, which under-sialylate the parasite proteins. This might suggest its usage as an anti-macrofouling and antifeedent agent [196,197]. Another cytotoxic macrolide that has been isolated from marine *Micromonospora* sp. strain L-25-ES25-008 is IB-96212. This macrolide elucidates eminent antimicrobial activity against *Micrococcus luteus* with an MIC score of 0.4 μg/mL [198]. 

The lomaiviticins A and B secreted by halophilic *Micromonospora* sp. strain LL-37I366 that harness two diazotetrahydrobenzo[b] fluorene (diazofluorene) functional groups are strong antibiotics that can cause double-strand breaks in the genome of eukaryotic cells [199]. *Micromonospora* sp. strain CA-214671 organic culture extracts are affluent with spirotetronate phocoenamicins, phocoenamicins B and C. Both compounds exert acceptable antibacterial effects toward Gram-positive microorganisms (MRSA, *B. subtilis*, vancomycin-resistant *E. faecium* (VRE)) with MIC scores ranging from 4 to 8 µg/mL [200]. Akazaoxime, a novel erythromycin-class antibiotic, is retrieved from marine *Micromonospora* sp. strain A-76356 cultures. Akazaoxime harbors an aldoxime moiety instead of O-methyl nitronic acid, and the carbon skeleton itself is composed of leucine, glycine, and propionate (methylmalonate). Akazaoxime elucidates mild antibacterial activity against the Gram-positive pathogen *Kocuria rhizophila* with an MIC score of 50 μg/mL. The synthetic counterparts of akazaoxime antibacterial performance were tested against the human pathogen *Trichophyton rubrum* and the plant pathogen *Glomerella cingulate*, and the reported MIC values laid within the range of 25–50 μg/mL [200,201]. Interestingly, *Micromonospora marina* is able to produce a rare 15-membered ring diterpene alcohol called micromonocyclol due to the presence of a unique terpene synthase [202,203]. Table 4 summarizes the chemical formula, structure, and mechanisms of *Micromonospora* sp. antifouling and antibacterial agents.

#### 5.2.2. Antifouling Agents from *Nocardia* sp. Genera

##### *Nocardia* sp. Terpenoids and Furanone

The marine actinomycete *Nocardia* sp. strain ALAA 2000 collected from red alga *Laurenica spectabilis* secrete various potent antimicrobial agents. Their extracts include chrysophanol 8-methyl ether, ayamycin, 1,1-dichloro-4-ethyl-5-(4-nitro-phenyl)-hexan-2-one, dichloro-4-ethyl-5-(4-nitro-phenyl)-hexan-2-one (also known as ayamycin), asphodelin, bichrysophanol, and justicidin B. Asphodelin and chrysophanol 8-methyl ether contain peri-hydroxy-anthraquinones. The chrysophanol 8-methyl ether is also known for having chelated OH as well as aromatic methyl and methoxy groups. Asphodelin (Table 4) resembles the microcarpin 2,7-bichrysophanol and cassiamin C. While ayamycin has an uncommon combination of a 1,1-dichloro moiety together with a nitro aromate moiety, it strongly mimics the chloramphenicol structure. On the other hand, justicidin B (Table 4) contains an arylnaphthalene moiety. Chrysophanol 8-methyl ether, asphodelin, bichrysophanol, and justicidin B have varying antimicrobial activities with MIC values ranging between 0.1–10 μg/mL against Gram-positive (*Bacillus cereus*, *Staphylococcus aureus*, *Micrococcus luteus*), Gram-negative bacteria (*Escherichia coli*, *Pseudomonas aeruginosa*), and fungi (*Rhodotorula acuta*, *Pichia angusta*, *Cryptococcus neoformans*, *Candida albicans*, *Aspergillus niger*, and *Botrytis fabae*) [207,208,209].

##### *Nocardia* sp. Alkaloids 

Novel indolocarbazole alkaloids loonamycin A–C are retrieved from the marine-derived *Nocardiopsis flavescens* strain NA01583. The presence of 18 olefinic carbons, 8 oxygenated methines, 2 oxygenated methylene carbons between 60 and 100 ppm, 1 methylene, and 4 methyl groups are indicated by the 13C NMR investigations of loonamycin A. The N-methyl singlet proton signal appearing within the 1H, 1H1H COSY, and HSQC NMR spectra at 3.22 ppm that correlates with HMBC, showing two carbonyl carbons at 168.8 and 173.9 ppm, respectively, indicates the presence of a N-methyl maleimide moiety. Combining these findings, an indolocarbazole core with two distinctive pyranohexose moieties was proposed. Loonamycin B is a N6-desmethyl derivative of loonamycin A. There is a striking resemblance between the indolocarbazole skeleton of loonamycin and staurosporine and rebeccamycin. Loonamycins are potent cytotoxic agents with IC50 scores ranging from 41 to 283 nM and have the ability to inhibit the notch signaling pathway [210,211]. Nocarbenzoxazoles A–G purified from *Nocardiopsis lucentensis* DSM 44048 cultures have negligible to no bioactivity except for nocarbenzoxazoles G, which demonstrate selective cytotoxicity towards HepG2 and HeLa with IC50 scores of 3 and 1 μM, respectively [212]. 

Two polycyclic thioalkaloides, dassonmycins A and B, were found among the organic extracts of actinomycete *N. dassonvillei* SCSIO 40065. Both compounds harbor a naphthoquinone [2,3-e] piperazine-[1,2-c] thiomorpholine skeleton. They are antibacterial when tested on *Micrococcus luteus*, *MRSA*, *B. subtilis*, and *S. aureus*, reporting MIC scores within 8–64 μg/mL. Further, dassonmycin B demonstrates antibacterial vigor toward *Vibrio alginolyticus* and *Enterococcus faecalis* with an average MIC score of 32 μg/mL [213]. Two novel compounds 2-hydroxyacetate-3-hydroxyacetamido-phenoxazine and questiomycin A are retrieved from marine *N. dassonvillei* JS106 cultures and manifest antiquorum sensing activities against *Staphylocccus aureus* and *Pseudomonas aeruginosa* [214]. The nocarterphenyls D-H prevalent in the cultures of actinobacterium *Nocardiopsis* sp. HDN154086 manifest antibacterial potentiality against *E. coli*, *Proteus* sp., *M. phlei*, *V. parahemolyticus*, *B. subtilis*, *B. cereus*, and *MRSA* [215]. Table S3 summarizes the chemical formula, structure, and mechanisms of *Nocardia* sp. antifouling and antibacterial agents [212,214,216,217,218].

##### *Nocardia* sp. Polyketides 

The *Nocardiopsis* sp. strain HB-J378 isolated from the marine sponge *Theonella* sp. generates the polyketides nocardiopsistins A–C. Nocardiopsistin A comprises an angucyclinone skeleton, which resembles an oviedomycin. Nocardiopsistin B is similar to its opponent A except for the presence of a methylene group and a quaternary carbon with the loss of two olefinic resonances. Nocardiopitin C is similar to nocardiopsistin B except for an extra methylene group and the removal of the resonance caused by the C-4 ketone. Nocardiopitin B exerts the uppermost anti-MRSA activity with the lowest MIC score of 3.12 μg/mL, resembling the positive control agent chloramphenicol. The three compounds did not show any antifungal activity against *C. albicans* sp. [215,219]. *Nocardiopsis* sp. HB-J378, which acquired a brominase-containing biosynthetic gene cluster known as *ncd*, generates the novel brominated nocardiopsistin D along with two sulfur-containing nocardiopsistins E-F. With MIC scores of 0.098, 3.125, and 0.195 g/mL, nocardiopsistin D, E, and F attain anti-MRSA activity, respectively. The single bromination in nocardiopsistin D significantly elevates the anti-MRSA performance by 128-fold and aids its antibacterial vigor towards vancomycin-resistant *S. aureus* (VRSA), *Enterococcus faecium*, and *Bacillus cereus* [220].

Marine *Nocardiopsis dassonvillei* subsp. *dassonvillei* DSM 43111 generates a novel polyketide known as nocapyrone S. Nocapyrone S acquires a carbonyl, two trisubstituted double bonds, an oxygen quaternary carbon, two methines, a methylene, and four methyls in its ^1^H and 13C NMR spectra, which indicates the presence of a α-pyrone moiety. The *Nocardiopsis dassonvillei* subsp. *dassonvillei* DSM 43111 also secrete (4-aminophenyl) acetic acid, N-(2-hydroxyphenyl)-acetamide, cyclo-(L-Pro-L-Val), cyclo-(L-Pro-L-Leu) and cyclo-(L-Pro-L-Ile). The extracts of *dassonvillei* DSM 43111 are cytotoxic [220]. The organic extracts of *Nocardiopsis* strain HB383 associated with the marine sponge *Halichondria panacea* contained γ-pyrones nocapyrones A-D. Nocapyrones A and B failed to inhibit phosphodiesterase 4, protein tyrosine phosphatase 1B, acetylcholinesterase, reverse transcriptase, or glycogen synthase kinase 3 and were deprived of any Gram +ve or Gram –ve antibacterial activity. The strain HB383 produces 2-[(4-methoxyphenyl)methylene]-5-(2-methylpropylidene)-3,6-piperazinedione with a diketopiperazine skeleton. These compounds are cytotoxic [221]. Deep-sea derived actinomycete *Nocardiopsis* sp. HDN 17–237 yields two novel compounds: α-pyrone nocapyrone T and β,γ-butenoate derivative phenylbutenote. Despite their novelty, both compounds failed to exert any antibacterial activity [218]. 

#### 5.2.3. Antifouling Agents from the Minor Families *Pseudonocardiaceae* and *Glycomycetaceae*

The glycosylated polyol macrolide aculeximycin (Figure S1) secreted from *Kutzneria albida* is an enormous oligosaccharide-macrolide that possesses a 30-membered polyhydroxy macrocyclic lactone and five sugars (aculexitriose, trisaccharide aculexitriose, L-vancosamine, and D-mannose) [222]. This compound acquires vigorous larvicidal and antimicrobial performances when tested against mosquito larvae, Gram-positive and Gram-negative bacteria, yeasts, and molds [223,224]. The 46 genome clusters responsible for the expression of aculeximycin are also prevalent among *Kutzneria viridogrisea*, *Kutzneria albida*, *Kutzneria kofuensis*, *Kutzneria buriramensis*, and *Kutzneria chonburiensis* [224]. Epemicins A and B are novel 30-membered glycosylated macrolides that resemble aculeximycin. Both compounds are retrieved from the cultures of the rare organism *Kutzneria* sp. that harbors the *BGC* gene cluster. Epemicins A and B possess anti-MRSA activity with MIC scores of 2−4 μg/mL and 1−2 μg/mL, respectively [225]. The organisms belonging to the *Pseudosporangium* genus generate novel oligomycin-class polyketides called pseudosporamicins A–C. Pseudosporamicins A–C manifest good antibacterial activity against the Gram-positive bacterium *Kocuria rhizohpila* [226]. Novel antibiofilm diketopiperazines are opulent among marine *Glycomyces sediminimaris UTMC 2460* culture extracts. UTMC 2460 diketopiperazines demonstrated anti-microfouling activity against marine fouling of *Kocuria* sp. and *Mesorhizobium* sp. [227]. 

## 6. Obstacles Facing Commercial Use of Natural Antifouling Agents 

A significant barrier to converting marine natural chemicals into commercial goods has always been the question of supply. As previously discussed, the extracts from marine actinomycetes represent promising antibacterial and antifouling agents; however, the yield is relatively low, and the retrieved volumes are usually not suitable for commercial usage. Thus, chemical synthesis studies should be expanded to facilitate the production of natural AF agents on larger scales. For instance, laboratory-produced monoterpene–furan geraniol hybrid molecules effectively suppressed cypris larvae of the barnacle *Balanus Amphitrite* more than the natural parent did [228]. 

The late-stage divergent method is used to formulate hybrid compounds of geraniol and butenolide. The butenolide moiety was built by ring-closing metathesis, and the eight synthetic hybrid compounds were biologically assessed. The synthetic hybrids attain anti-macrofouling activities against *Balanus Amphitrite* cyprid larva with EC50 values of 0.30–1.31 μg mL^−1^. This outcome paved the way for the successful hybridization of the geraniol and butenolide structural motifs that are associated with high anti-macrofouling activities [229]. 

Further, nine antifouling hybrid compounds were formulated and biologically tested by combining a dihydrostilbene scaffold with the oxime moiety prevalent among the structures of marine antifoulants. The generated hybrids exert aligicidal activity that prohibited microalgae settlement and proliferation with the best-performing hybrid recording an MIC score of 0.01 μg/mL [230]. In similar manners, 22 synthetic dihydrostilbenes hybrids were formulated with variable substitution patterns, and their antifouling capabilities were explored against 16 marine polluting organisms. The synthetic hybrids harnessing the dihydrostilbene scaffold elucidated acceptable anti-micro- and macrofouling profiles. The hybrids with the uppermost antifouling properties that were comparable to biocide Sea-Nine (positive control) were 3,5-dimethoxybibenzyl, 3,4-dimethoxybibenzyl, and 3-hydroxy-3′,4,5′-trimethoxybibenzyl [231]. 

The C24–C40 section of aculeximycin is stereo-selectively synthesized through epoxy-opening rearrangement events and Kobayashi aldol reactions. First, the C25–C32 segment was formulated by a Kobayashi aldol reaction followed by epoxidation and Jung rearrangement of epoxide 9. The other segment C33–C40 was formulated by a Kobayashi aldol reaction only. For the final step, both segments were fused by an adol reaction that converts ethyl ester to ethyl ketone followed by subsequent dehydration [232]. Justicidin B can be also laboratory hybridized through Suzuki–Miyaura cross-coupling of a triflated naphthalene lactone intermediate and various potassium organotrifluoroborates [209]. 

The second barrier before the development of AF compounds is the need for rigorous, reliable, and broad-spectrum bioassay systems in research labs, which is yet unfulfilled. Several marine organisms were used during the assessment of the anti-micro/microfouling agent activities for the compounds of interest (larva, algae, bacteria, fungi, etc.). A common feature observed among these assays was the variability of the test outcomes due to the different experimenting techniques that affect the overall reliabilities of the performed tests. It is a prerequisite to develop anti-microfouling and anti-macrofouling bioassay systems that include as many target species as feasible. A robust anti-macrofouling bioassay system should include sessile hard foulers that are regularly found in fouling communities, such as barnacles and tube-building worms, as well as soft foulers, such as the bryozoan *B. neritina* or seaweed, such as *Ulva*. Collaboration among research labs should be encouraged to overcome geographic constraints [118]. A study conducted by Gama et al. (2003) evaluated the effectiveness of laboratory mussel tests for the determination of the antifouling activity of extracts from the Brazilian seaweeds *Laurencia obtusa* and *Stypopodium zonale* versus in the field using the “phytagel method.” In both the laboratory and field trials, *L. obtusa* extracts greatly reduced fouling, whereas *S. zonale* increased fouling. Although field assays are preferred, the results indicate that the “mussel test” is a trustworthy time- and money-saving screening tool for antifouling chemicals [233]. 

## 7. Prospects for the Optimization of Antifouling Agent/Natural Bioactive Molecule Production with Marine Actinomycetes

Marine actinomycetes, as previously mentioned, are intriguing sources of naturally occurring antifouling agents that showed equal activities to those of traditional chemical antifouling agents with acceptable LC50/EC50 profiles [130]. However, in order to achieve maximum production and activity, the yield of antifouling agents by marine actinomycetes is largely reliant on growth conditions. Therefore, optimization techniques must be used before scaling up bioactive material production. In order to guarantee the optimization process’ relevance and dependability, it is also necessary to strengthen it with the best statistical design [118]. 

By adjusting the concentrations of both carbon and nitrogen sources, Sebak et al. (2021) optimized the production of antimicrobial agents secreted by *Streptomyces* sp. cultures for the utmost yield and activity of antimicrobial agents. In their research, different mono-, di-, and polysaccharide sugars (fructose, lactose, glucose, etc.) were used to substitute starch as a carbon source in ISP4 broth. Ammonium sulfate, urea, peptone, tryptone, proteose peptone, albumin, casein, casamino acid, and yeast extract were also nitrogen source substituents within the ISP4 broth. The *Streptomyces* sp. cultures were then fermented for 11 days with continual sampling to select the perfect growth conditions and best C/N sources. The influence of an extracting solvent on the potency of antimicrobial/antifouling agents has been also explored by using dichloromethane (DCM) and ethanol (EtOAC). Sebak et al. (2021) denoted that maltose and casein are the optimal carbon and nitrogen sources, respectively, for the *Streptomyces* sp. *extract* to exhibit the largest inhibitory zone on different bacterial species. DCM (1:1, *v*/*v*) is the best extraction solvent; however, EtOAc in a higher solvent-to-broth proportion (2:1, *v*/*v*) revealed the same result. This research demonstrated that a *Streptomyces* sp. antibacterial extract rich in hydroxylated fatty acids may be enhanced by optimization, which facilitated the scaled-up manufacture of antimicrobial/antifouling agents [234].

To elicit antimicrobial/antifungal agents’ production by marine *Streptomyces* sp. MK388207 (M12) cultures, Hamed et al. (2019) deployed a seven-variable Plackett Burman optimization design. These variables included the medium C source, the concentration of mineral salts, the pH, and the incubation duration. According to their research, starch, MgSO_4_.7H_2_O, and FeSO_4_, all have positive influences on *Streptomyces* sp. *MK388207* antimicrobial agent yield and activity. However, other mineral salts (KNO_3_, k_2_HPO_4_) have a negative impact on the yield and performance of antimicrobial metabolites. Additionally, the study has reported that leveling up the incubation temperature is accompanied by enhanced antibacterial activity, but beyond 35 °C, this effect is reversed. The study demonstrated that enhanced production of bioactive compounds from *Streptomyces strain MK388207* could be successfully attained by optimizing the fermentation cultures [235].

Similar techniques were used by Hassan et al. (2017) to maximize the synthesis of enterocin, a powerful antibacterial agent against *Listeria* sp., with the marine actinobacterium *Streptomyces* sp. *H-1003* [236]. The final optimization procedure necessitated the cultivation of the marine actinomycte for 10 days with a starch-amended Gause’s medium (20 mg/L) and cobalt ions (2 mM). Under these conditions, enterocin production peaked at 5.33 mg/L, which was much higher than other observed metal stress conditions. It was also noted that enterocin was absent among H-1003’s non-optimized cultures. This study denoted that heavy metals can elicit the production of enterocin and other bioactive compounds from *Streptomyces* sp. [236,237]. Similarly, *Streptomyces* sp. 891, a wild sea strain, generates chrysomycin A, an antibacterial agent that is effective against both MRSA and resistant *Tuberculosis* strains, under ideal optimized conditions. The research suggests that for a 168 h incubation period, the optimum growth media should comprise CaCO_3_ (3 g/L), hot-pressed soybean flour (25 g/L), maize starch (20 g/L) with glucose, as well as a 5% inoculum size (48 h incubation). Optimal growth conditions enhanced the chrysomycin A yield by approximately a fivefold factor. These outcomes unquestionably paved the way for chrysomycin A preparation to be produced in greater quantities and used in the pharmaceutical industry [238].

By altering the carbon and nitrogen sources and mineral salts in the fermentation media, it was also possible to optimize the synthesis of platensimycin (PTM) and platencin (PTN), two powerful antibacterial agents identified from different strains of *Streptomyces platensis*. The maximum titer of PTM was produced with a specifically developed medium that is opulent with starch, soybean flour, MOPS sodium salt, and CaCO_3_. The 60 L scale-up fermentation successfully produced 45.14 g of PTM. These results would speed up industrial PTM’s development for its potential use as an antibacterial agent [239]. Using an eight-variable Plackett–Burman design and response surface approach, the synthesis of numerous enzymes, including the l-asparaginase, was also carried out using *Streptomyces koyangensis SK4* bacterial cultures. Temperature, pH, incubation time, agitation rate, and asparagine concentration were found to have a substantial impact on the production of l-asparaginase. On the seventh day of incubation, the asparagine dextrose broth that was kept at a pH of 7.5, shaken at 125 rpm, and supplemented with l-asparagine (7.5 g/L) attained the maximal enzyme activity of 136 IU/mL. The arctic actinomycetes produced high l-asparaginase yields thanks to the statistical optimization technique [240]. 

Using 16 variable Plackett–Burman statistical designs, the production of L-asparaginase was also leveled up from *Streptomyces parvus NEAE-95* cultures. Several factors affected the production of L-asparaginase, including the incubation time, L-asparagine, and yeast extract. The optimal levels of these crucial variables and the influence of their interactions were achieved using a Box–Behnken statistical design [241]. Further, El Naggar et al. (2019) improved L-asparaginase activity from *Streptomyces brollosae* NEAE-115 broth culture following a 96 h shaking incubation period (400 rpm) with a broth media affluent with extrose (2 g), starch (20 g), L-asparagine (10 g), KNO_3_ (1 g), K_2_HPO_4_ (1 g), MgSO_4_.7H_2_O (0.5 g), and NaCl (0.1 g) at a neutral pH [242]. 

Genetic modification tools can also be utilized to optimize the production of antifouling agents and various secondary metabolites from marine actinomycetes. She et al. (2022) regulated the production of albofungin through a genetically modified *Streptomyces* strain that can overexpress the transcription regulators (*Alb22* and *alb45*). The transcription regulators were digested from template *Streptomyces chrestomyceticus* DNA (previously exhibited an eminent ability for albofungin production) and cloned to generate plasmid constructs (24770/pPWW-alb22 and 24770/pPWW-alb45). The plasmids were then conjugated into *S. chrestomyceticus BCC 24770*, and the isolates were grown on a selective plate (apramycin and nalidixic acid as antibiotic markers) to isolate the positive conjugate. Interestingly, the albofungin yield was enhanced following the introduction of the *alb22* and *alba45* constructs while maintaining its antibioflim, anti-macrofouling, and antibacterial activity. The present study offers a new genetic prospective for the production of antifouling agents and elevated the yield and biological activity of albofungin via the overexpression of *alb22* and *alb45* activators [186]. Cloning the novel type II polyketide synthase pathways into *Streptomyces albus J1074* was also associated with favorable yields of a brand-new antibacterial agent, tetarimycin A. The genetic modification facilitated the introduction of the *ermEp* promoter before the putative *SARP* gene within *S. albus* J1074 DNA harboring the silent biosynthetic PKS cluster [243]. Further, the marine *Actinoalloteichus* sp. AHMU CJ021 harbored silent biosynthetic gene clusters (BGCs) that encoded the secondary metabolite of diverse biological activities, caerulomycin A (CRM-A). To initiate the expression of caerulomycin A, a ribosome engineering technique was applied to wild-type *Actinoalloteichus* sp. Using UV radiation-induced mutagenesis, the wild strain was bred with a mutant strain that could produce CRM with a 42.51 ± 4.22 mg/L titer. The levels of CRM-A were elevated post-UV exposure due to increased levels of intracellular riboflavin that created a new mutant strain entitled XC-11GUR with a CRM A production titer of 113.91 ± 7.58 mg/L. The new mutant strain was further optimized with different media compositions, and the yield was enhanced by 14.6-fold [244]. 

Zhang et al. (2023) engineered an *E. coli* strain that can express the powerful antifouling zosteric acid (ZA) formerly retrieved from seagrass *Zostera* sp. utilizing both glucose and glycerol. Interestingly zosteric acid elucidates eminent antifouling potential with minimal toxicities and high biodegradable abilities, yet the *Zostera* sp. yield of zosteric acid is very low. The *E. coli* strain was genetically manipulated to overexpress the gene’s needed for 3′-phosphoadenosine-5′-phosphosulfate (PAPS) production while knocking down the *cysH* gene responsible for PAPS consumption. The ZA-producing *E. coli* strain with an increased PAPS supply was further optimized by allowing the co-expression of sulfotransferase 1A1, tyrosine ammonia-lyase, adenosine 5′-phosphosulfate kinase, and ATP sulfurylase genes. Further, to overcome the negative feedback control initiated by L-tyrosine production, the feedback-resistant genes encoding 3-deoxy-D-arabinoheptulosonate 7-phosphate synthase and chorismate mutase were overexpressed, and the regulator genes, *tyrR* and *pheA*, were knocked out. Consequently, the strain with the best performance produced 1.52 g L^−1^ of ZA and 1.30 g L^−1^ of p-hydroxycinnamic acid. This technique can be applied to the production of low-yield antifouling agents using genetically modified *E. coli* strains [245]. 

## 8. Implementation of Novel Antifouling Bioassays to Overcome the Lack of In Vitro Test Reliability 

Prior to scaling up the production of natural antifouling agents, the ecologically friendly coating must be examined thoroughly with in vitro laboratory assays to confirm its antifouling activity. Therefore, reliable flow-through tests for antifouling agent coating-containing biocidial agents are crucial. Pansch et al. (2017) developed a flow-through bioassay that facilitates the assessment of low-release biocide AF paints and their impact on barnacle pre- and post-settlement features. The proposed bioassay can efficiently distinguish between bulk-water effects on biocide release and direct surface contact biocide release. This is crucial during the creation of low-emission AF coatings. Briefly, the bioassay was based on a flow cell that elucidated the capability of the biocide to halt barnacle larvae (cyprids) settlement. The cell harnessed a V-shaped section where two panels were fixed face-to-face at 120° degrees. The inner stands within the flow cell were covered by a plankton net that allowed the passage of water without disrupting the cyprids. The inside of the cell was also covered with a nylon net that hindered the cyprid settlement in any undesired areas. The architect of the flow cell ensured that only the treatment panels were accessible by the cyprids for settlement and the assessment of the anti-macrofouling capability of the biocide. To address the issue of biocide release into the bulk water, experiments could be held with two flow cells, upstream and downstream cells, where the downstream cell was fed by the output retrieved from the upstream cell and thus acted as a control panel for testing the biocide released from the coating surface. This was tested using a copper-based antifouling coat where inhibition of barnacle settlement occurred in the downstream cell, suggesting their frequent release into bulk water. The most advantageous feature of these cells is that they allow easy manipulation of the test factors for a better understanding of antifouling mechanisms [246]. 

In similar manners, Kojima et al. (2019) utilized a flow-through laboratory anti-*B. amphitrite* larval settlement assay using triangular boxes. Here, the triangular boxes were put together in tanks containing the seawater test samples one day earlier to the bioassay’s conduction and left overnight at 25 °C. The boxes were then removed and submerged again for five minutes in 1 L of seawater before the bioassay. The top side of the triangle box was opened to allow the passage of saltwater into the bioassay tank. Each of the triangular boxes comprised 100 cyprids, and the test seawater, pH, and salinity levels were regularly monitored. The system was advantageous in many ways since it had a low flow rate, and the density of the cyprids was constantly adjusted. Also, the assay was solely dependent on how well the test antifouling coating worked where it employed an inert white acrylic plate coated with AF agent and trapped the cyprids inside a triangular vessel, which increased cyprid settlement on the surface of the coated plate only. By the end of the bioassay, the unattached cyprids and dead individuals inside the triangular box were promptly collected, and the triangular boxes were disassembled. Under a stereo microscope, the number of juveniles, cyprids, and deceased individuals on each of the triangle box’s three surfaces as well as on the plate were tallied. This method is novel in that it evaluates the behavior of barnacles inside a triangular box in a flow-through system to determine how well antifouling paints inhibit barnacle growth. This study also demonstrated a highly reliable way for antifouling agent efficacy evaluation [247]. 

Antunes et al. (2019) designed a multi-bioassay approach that efficiently assessed the potential antifouling activities of cyanobacterium *Phormidium* sp. portoamides while including the associated marine toxicities and exploring the molecular mode of action. In their work, they utilized several bioassays. The *Mytilus galloprovincialis* mussel larvae anti-settlement bioassays were incorporated to assess whether the AF impact of portoamides is acute and reversible or long-lasting. Conventional antibacterial, antifungal, and antibiofilm tests were also incorporated; however, a quorum-sensing inhibition (QSI) quantitative assay (violacein inhibition assay) was performed to quantify the portoamides’ ability to halt QS. Finally, a marine eco-toxicity assay was performed using the *Artemia salina nauplii* lethality test. The study denoted that the portoamides revealed broad spectrum antifouling activity with an anti-mussel settlement EC50 value of 3.16 μM due to the inhibition of proton-transporting ATPases activity and the induction of gill alterations within the mussels. The AF activity of the portoamides was also non-toxic and reversible, suggesting its potential usage as a natural antifouling coating agent [248]. 

## 9. Future Perspectives 

The manufacturing of natural AF agents on greater scales should be made possible by expanding chemical synthesis and production optimization research to enable their commercial usage. The response surface methodology (RSM) using multifactorial design should be implemented for the best production of various secondary metabolites, including antibacterial, [190,249], antifungal, [250,251], and biosurfactant metabolites [252,253]. It has been previously established that growth condition statistical optimization improves the yield and the activity of various secondary metabolites. Therefore, further studies are still required to improve the production of natural antifouling agents. Research into the molecular mechanisms of action for natural antifouling compounds is necessary in order to provide a thorough and in-depth explanation for their inhibitory pathways. The production of various natural AFs is now available without the burden of isolating marine actinomycetes from their original habitat through the utilization of various genetic modification techniques (protoplast fusion, cloning, homologous and heterologous expression, etc.). Genetic manipulations facilitate the expression of the active metabolite via various laboratory expression vectors (e.g., *E. coli* strains) with a high yield and facilitate easy optimization. This will revolutionize the commercial industry of natural antifouling agents. To further understand the real biocidal action of these antifouling chemicals, investigate any potential toxicity, and determine whether species have developed antifouling resistant mechanisms against these antifouling agents, field investigations in oceans and seas must be implemented and expanded in different areas worldwide. Exploring antifouling agent resistance among fouling bacterial species and other organisms during field investigations will be substantially eased by the introduction of various biosensors [254].

## 10. Conclusions

This review emphasized the negative effects of biofouling (medical, marine, or industrial) and the implemented strategies to overcome it. Considering that the largest untapped source of natural goods is still marine microorganisms, a wide variety of novel actinomycetes strains were recovered from different marine environments over the past years. The *Micromonosporaceae*, *Nocardioidaceae*, *Pseudonocardiaceae*, and *Streptomycetaceae* actinomycetes families were home to the organisms that were most frequently isolated from the marine sediments. The collected marine organisms were profitably and effectively utilized to extract brand-new compounds that belonged to a variety of antifouling agent classes. These compounds have shown positive anti-microfouling and anti-macrofouling properties that will enable their application as marine and medicinal antifouling agents for future applications. To provide a complete and detailed description of these novel antifouling agents, research on the molecular mechanisms of action of these antifouling compounds needs to be conducted. Different optimization techniques should be implemented to improve the yield of natural antifouling agents with actinomycetes. The introduction of genetic modification techniques will revolutionize the industry of natural antifouling agents.

## Figures and Tables

**Table 1:** Natural antifouling agents, secreting organisms, mode of action, and sources.

Natural AF Class	AF Agent	Secreting Organism	Mode of Action	EC50 Value	References
Terpenoids and steroids	Avarol and avarone, avarone derivatives (Chlorophenylavarone, Ethylenedithioavarone, Isopropylthioavarone, tert-Butylthioavarone, Propylthioavarone, Octylthioavarone)	Mediterranean sponge *Dysidea avara*	Anti-macrofouling vigor toward *Balanus amphitrite* cyprids Antibacterial activity against larval settlement inducers (*C. marina*, *M. stanieri*, *V. fischeri*, *P. haloplanktis*) Antifunagl activity (*A.cruciatus*, *L. uniseptata*)	EC50s (μg/mL) Avarol: 0.65 Avarone: 3.41 Chlorophenylavarone: 0.65 Ethylenedithioavarone: 26.22 Isopropylthioavarone: 1.33 tert-Butylthioavarone: 4.23 Propylthioavarone: 0.45 Octylthioavarone: 1.46	[106]
	Isocyanate and isothiocyanate derivatives of kalihinane diterpenes	Marine sponge *Acanthella cavernosa*	Anti-macrofouling vigor toward *Balamis amphitrite* cyprids	0.05 μg/mL	[107]
	Lobocompactol diterpenes	Brazilian brown alga *Canistrocarpus cervicornis* Marine actinomycete *Streptomyces cinnabarinus* PK209 cultivated in association with marine-derived *Alteromonas* sp. KNS-16	Inhibited mussel settlement Aligicidal action (macroalga *Ulva pertusa* and *Navicula annexa*, *diatom*)	For the larval settlement: 0.18 μg/mL For *Ulva pertusa* and the diatom *Navicula annexa*: 0.43 μg/mL	[108]
	Meroditerpenes	Mediterranean brown alga *Halidrys siliquosa*, Brown alga *Cystoseira foeniculacea*	Antifouling against *Balanus*, *amphitrite* cyprids.	<5 μg/mL	[109,110]
	Dihydrofurospongin II	Mediterranean *Spongia officinalis*	Antibacterial and antifungal activity Weak settlement inhibitory effect on barnacle larvae	100 μg/mL	[21,111]
	Agelasimines A and B, Agelasine D	Marine sponges *(Agelas* sp., *Raspailia* sp.)	Antimicrobial activity Strong inhibitory effect on settlement of Balanus improvisus cypris larvae	Agelasine D EC50 = 0.11 μM	[112]
	Cavernosolide and lintenolide A	New Zealand sponge *Senitaspongia bactriana*	Antifouling vigor towards *Nitzschia closterium* and *Bugula neritina*	Cavernosolide EC50 = 5.24 μM (*N. closterium*) and EC50 = 1.22 μM (*B. neritina*) Lintenolide EC50 = 6.72 μM (*N. closterium*) and EC = 1.59 μM (*B. neritina*)	[113]
Fatty acids	Palmitic acid	Brown alga *Sargussum muticum*	Inhibit the emergence of *Ulva* (green alga) spores	3 μg/mL	[114,115]
	2-Hydroxymyristic acid (HMA) and oleic acid (COA)	Marine bacterium *Shewanella oneidensis*	Blocked the germination of *Ulva pertusa* spores	10 and 100 µg/mL	[116,117]
	12-methylmyristic acid	Actinomycete *Streptomyces* sp.	Potent anti-larval activity against *Hydroides elegans*	0.625–40 μg/mL	[118,119]
	(3R,5S)-3,5-Dihydroxydecanoic acid	Marine-derived fungus *Aureobasidium* sp.	Antibacterial activity against *Bacillus subtilis*, *Escherichia coli*, and *Staphyllococcus aureus*	-	[120]
Amino acids and related compounds	Bastadins (bromotyrosine derivatives)	*Lissodendoryx isodictyalis* spong	Antifouling agent against *Balanus* *amphitrite*	<250 μg/mL	[121]
	Barettin and 8,9-dihydrobarettin (brominated diketopiperazines (DKPs))	Marine sponge *Geodia baretti*	Anti-macrofouling vigor against *Balanus improvisus*	0.9–7.9 μM	[122]
Alkaloids	Benzonaphthyridine	Marine sponge *Aaptos aaptos*	Inhibition of α_2_-adrenoceptors	-	[123]
	Araguspongine C	Indian marine sponge *Haliclona exigua*	Inhibited cypris settlement of *Balanus amphitrite*	6.6 μg/mL	[124]
	Camptothecin	Marine plant *Camptotheca acuminata*	Inhibited the cyrips larval settlement of bryozoan *Bugula neritina* and the barnacle *Balanus albicostatus*	EC50 (Bryozoan bioassay) = 43.11 μM EC50 (barnacle inhibition) = 4.97 μM	[125]
Benzenoids	Amibromdole	Soft coral-derived fungus *Sarcophyton* sp.	Weak AF activity against *B. amphitrite* larvae	16.70 μg/mL	[126]
	Luteolin-4-glucuronide	Seagrass *Enhalus acoroides*	Potent inhibitor of larval settlement of *Bugula neritina larvae*	0.52 μg/mL	[127,128]
Quorum-sensing inhibitors	Alkylated butenolides	Actinomycete *Streptomyces* sp.	Anti-macrofouling vigor larvae of *Balanus amphitrite*	5.43–12.4 μM	[105]
	Floridoside [a-D-galactopyranosyl-(1/2)glycerol]	Red alga *Galdieria sulphuraria*	Larval settlement of *Balanus amphitrite*	-	[129]

**Table 2:** Terpenoids retrieved from marine *Streptomyces* sp.

Compound	Structure, Chemical Formula, and MWT	Producing Organisms	Biological Activity	Reference
Oxycyclo-pentadien	C_5_H_6_O, Mwt: 82.10 g/mol 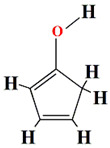	*Streptomyces thermolineatus* VITKV6A	Anti-microfouling, Anti-macrofouling, Non-toxic/eco-friendly	[136]
Napyra-diomycin A1	C_25_H_30_C_l2_O_5_, Mwt: 481.4 g/mol 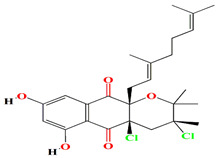	*Streptomyces* sp. YP127	Nrf2-activating efficacy, anti-oxidant, anti-inflammatory agent	[145]
Napyra-diomycin B3	C_25_H_29_BrCl_2_O_5_, Mwt: 560.3 g/mol 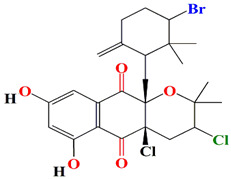	*Streptomyces* sp. SCSIO 10428	Antibacterial (Gram +ve bacteria), cytotoxic to cancer cell lines	[146]
Napyra-diomycin B	C_24_H_26_O_7_, Mwt: 426.5 g/mol 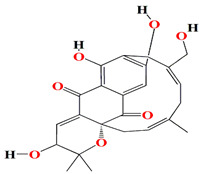	*Streptomyces* sp. strain CA-271078	Anti-infective and cytotoxic properties	[147]
4-dehydro-4a-dechloronapyradiomycin A1	C_25_H_29_ClO_5_, Mwt: 444.9 g/mol 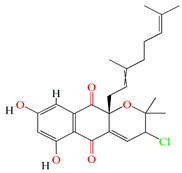		Potent antimicrobial activity	[148]
Naphterpin	C_21_H_22_O_5_, Mwt: 354.4 g/mol 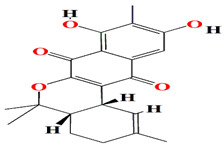	*Streptomyces* sp. CNQ-509	Anti-inflammatory	[140,149]
Nitropyrrolin A	C_19_H_30_N_2_O_4_, Mwt: 350.5 g/mol 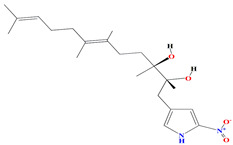		Weak antibacterial agent, cytotoxic	[150]
Marinone	C_25_H_27_BrO_5_, Mwt: 487.4 g/mol 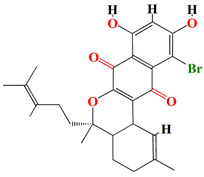	*Streptomyces* sp. CNQ-509	Antibacterial	[151]
Furaquinocin C	C_22_H_26_O_5_, Mwt: 370.4 g/mol 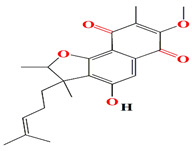	*Streptomyces* sp. KO-3988	Antibiotics	[152]
Furaquinocin D	C_22_H_26_O_6_, Mwt: 386.4 g/mol 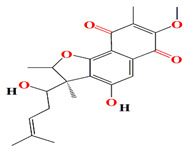			
Merochlorin B	C_25_H_29_ClO_4_, Mwt: 428.9 g/mol 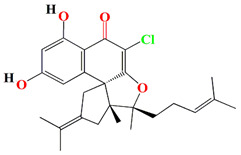	*Streptomyces* sp. strain CNH-189	Antibiotics	[153]
Merochlorin C	C_26_H_32_C_l2_O_5_, Mwt: 495.4 g/mol 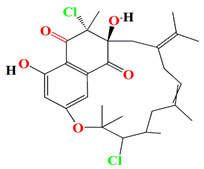			[154]

**Table 3:** Alkaloids retrieved from marine *Streptomyces* sp.

Compound	Structure, Chemical Formula, and MWT	Producing Organisms	Biological Activity	Reference
Strepchazolin A	C_12_H_17_NO_3_. Mwt: 223.27 g/mol. 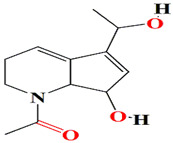	*Streptomyces chartreusis*	AChE inhibitory effect (neurotransmitter inhibitor, anti-macrofouling agent) Antibacterial (Gram +ve bacteria) Antifungal (pathogenic fungus *Escovopsis* sp.)	[177]
Streptazolin	C_11_H_13_NO_3_ Mwt: 207.23 g/mol 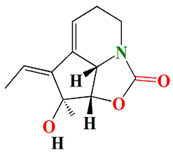	*Streptomyces* sp., *Streptomyces viridochromogenes*	Antibacterial (Gram +ve bacteria)	[178]
Streptoglutarimide A	C_15_H_21_NO_5_, Mwt: 295.33 g/mol 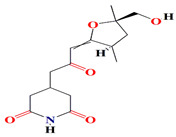	*Streptomyces* sp. ZZ741	Antibacterial and antifungal agents	[158]
Streptoglutarimide J	C_15_H_21_NO_4_, Mwt: 279.33 g/mol 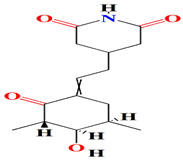			
Actiphenol	C_15_H_17_NO_4_, Mwt: 275.30 g/mol 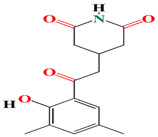	*Streptomyces pratensis*, *Streptomyces griseus*	AChE inhibitors, cytotoxic, antiviral	[159,179]
Streptimidone	C_16_H_23_NO_4_, Mwt: 293.36 g/mol 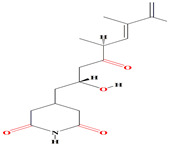	*Streptomyces* sp., *Streptomyces hygroscopicus*, *Micromonospora coerulea*	Antibiotic	[161]
Geranylpyrrol A	C_18_H_26_N_2_O_3_, Mwt: 318.411 Da 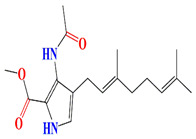	*Streptomyces* sp. CHQ-64	Cytotoxic activity	[159]
Anandin A	C_23_H_35_NO_2_, Mwt: 357.5 g/mol 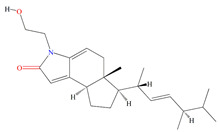	*Streptomyces anandii*	Antibacterial	[168,169]
Glucopiericidin C	C_30_H_45_NO_8_, Mwt: 547.7 g/mol 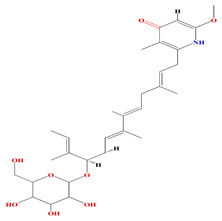	*Streptomyces* sp.	Antibacterial, cytotoxic activity	[180,181]
Malayamycin A	C_13_H_18_N_4_O_7_, Mwt: 342.30 g/mol 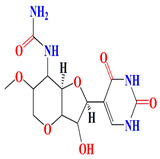	*Streptomyces malaysiensis*	Cytotoxic activity	[171]
Aranciamycin	C_27_H_28_O_12_, Mwt: 544.5 g/mol 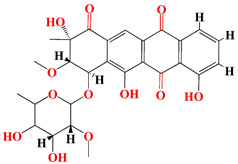	*Streptomyces* sp., *Streptomyces echinatus*	Antibiotic/antibacterial	[182]
Isotirandamycin B	C_22_H_27_NO_8_, Mwt: 433.5 g/mol 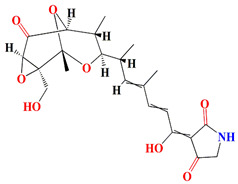	*Streptomyces* sp. *SCSIO 41399*	Bacteriostatic agent	[173]
Tirandamycin	C_22_H_27_NO_7_, Mwt: 417.5 g/mol 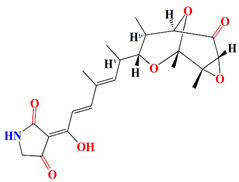			

**Table 4:** Antifouling/antibacterial agents retrieved from *Micromonospora* and *Nocardia* sp.

Compound	Structure, Chemical Formula, and MWT	Producing Organisms	Biological Activity	Reference
Diazepinomicin	C_28_H_34_N_2_O_4_, Mwt: 462.6 g/mol 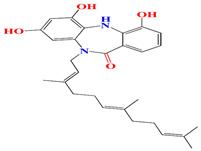	*Micromonospora* sp., *Micromonospora* strain DPJ12	Anti-protease, antimicrobial, antiparasitic	[204]
Megalomicin A	C_44_H_80_N_2_O_15_, Mwt: 877.1 g/mol 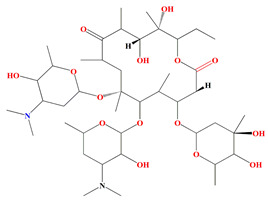	*Micromonospora megalomicea*	Antiparasitic, anti-macrofouling, antibacterial	[205]
Lomaiviticin A aglycon	C_38_H_26_N_4_O_14_, Mwt: 762.6 g/mol 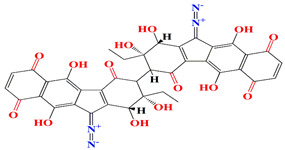	*Micromonospora* sp. strain LL-37I366	Antibiotics	[199]
Lomaiviticin B	C_52_H_50_N_4_O_20_, Mwt: 1051.0 g/mol 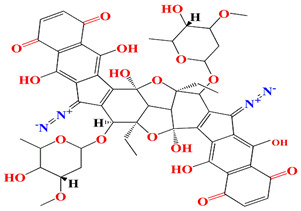		Antibiotics	[171,206]
Phocoenamicin B	C_56_H_75_ClO_19_, Mwt: 1087.6 g/mol 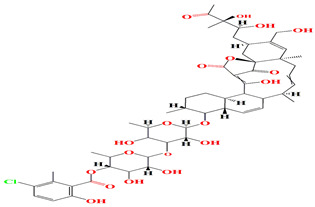	*Micromonospora* sp. strain CA-214671	Antibacterial (Gram +ve bacteria)	[200]
Ayamycin	C_14_H_17_C_l2_NO_3_, Mwt: 318.2 g/mol 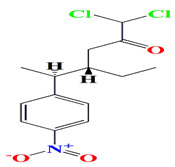	*Nocardia* sp. strain ALAA 2000	Antibacterial (Gram +ve MRSA)	[207]
Asphodelin	C_30_H_18_O_8_, Mwt: 506.5 g/mol 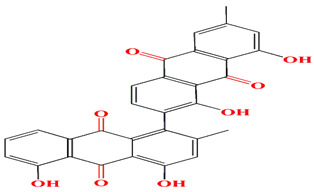		Antibacterial	[208]
Justicidin B	C_21_H_16_O_6_, Mwt: 364.3 g/mol 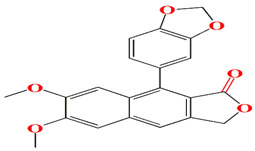		Antibacterial, antifungal	[209]

## Data Availability

Not available.

## Supplementary Materials

The following supporting information can be downloaded at https://www.mdpi.com/article/10.3390/microorganisms11102444/s1. Figure S1: Aculeximycin retrieved from *Kutzneria albida*, Table S1: Classification of marine/environmental fouling, Table S2: Commonly used synthetic medical/environmental antifouling coatings, Table S3: Alkaloids and polyketides retrieved from *Nocardia* sp.
