# Supplementary material for: New Insights on Biological Activities, Chemical Compositions, and Classifications of Marine Actinomycetes Antifouling Agents
Source: Microorganisms. 2023 Sep 29;11(10):2444. doi: 10.3390/microorganisms11102444 (PMC10609280; doi:10.3390/microorganisms11102444)
Supplement: Supplementary file 1 [file microorganisms-11-02444-s001.zip › microorganisms-2596424-supplementary.pdf]

Table S1. Classification of marine/environmental fouling

	Marine Fouling	
	**Microfouling**	**Macrofouling**
Causative agents	Bacteria (*Pseudomonas* sp., *Kocuria* sp. and *Mesorhizobium* sp.)<br>Proteins<br>Diatoms (e.g. *Amphora*, *Navicula*, *Nitzschia*)	Bryozoa (e.g. *Bugula neritina*)<br>Barnacles (e.g. *Balamis amphitrite*)<br>Mussels<br>Algae
Duration of contact	Seconds to minutes post-exposure minutes to hours (formation of biofilm slime)	Hours to Days
Settlements on the submerged surfaces	**1ry bacterial attachment:** physicochemical interactions (van-der-Waals,<br>electrostatic, hydrophobic, etc.)<br>**2nd bacterial attachment:** production of extracellular polymeric substances, formation of slimy biofilms<br>**Diatoms:** Attachment to extracellular polymeric substances, followed by permanent attachment through pads, stalks, tubes, or films	**Bryozoa:**<br>Acid mucopolysaccharide adhesive<br>**Barnacles:**<br>Cyprid (or adult) cement containing crosslinked o-quinones (highly rich in<br>serine, threonine, glycine, and alanine amino acids)<br>**Mussels:**<br>glycoproteins (cross-linked scleroproteins)<br>**Algae:**<br>Motile spores flagella and extracellular residual<br>mucilage present on the spore surface
**References**	[32-34]	[35-38]

Table S2. Commonly used synthetic medical/environmental antifouling coatings.

Type of fouling	Antifouling mechanisms/coatings	Reference
Medical Fouling	Ag, Se, Ti or Zn nanoparticles coated surfaces	[55-59]
	Hydroxyapatite and nanohydroxyapatite coatings	[60,61]
	Silane coatings, nanoplasma trimethyl silane	[62]
	Ceramics eg. calcium phosphate (long-term antifouling effect) and Mg-based implants	[63,64]
	Oligoethylene glycol and polyethylene glycol coatings (reduce deters fibrinogen adsorption on surfaces)	[65]
	Superhydrophilic Zwitterionic polymers	[66]
	Fluoropolymer and fluorosilane (high biocompatibility in implantable devices with reduced protein adsorption)	[67,68]
	Polyampholyte hydrogel grafted onto polyethersulfone membrane (low fouling surface coatings)	[69]
	Polythiourethane/ZnO-based anti-fouling materials	[70]
Marine/ Industrial fouling	Grapheme cuprous oxide or Silver-coated surfaces	[71,72]
	Silicone elastomer grafted with telomer of dodecafluoroheptyl methacrylate (DFMA), poly(ethylene glycol) methyl ether methacrylate (PEGMA), and 3-mercaptopropyl trimethoxysilane (KH590) to bis-silanol terminated silicone (eco-friendly fouling release materials)	[73,74]
	Polydimethylsiloxanes (PDMS) coatings	[75]
	SLIPS (slippery liquid-infused porous surfaces)	[76]
	PEG hydrogels and brushes	[77]
	Amphiphilic polymer coatings (polystyrene-polybutadiene-polystyrene (SBS), vinyl fluoride silicone (BD-FT-LSR), and PEG)	[78]
	TiO_2 and Ti-Cu-O Films	[79]
	Methoxy-terminated poly(ethylene glycol) (mPEG) conjugated to the adhesive amino acid l-3,4-dihydroxyphenylalanine (DOPA)	[80]
	Saccharide-Functionalized Alkanethiols	[81]
	Irgarol 1051 and Sea Nine 211	[82]
	Zinc pyrithione (ZnPT)	[83]

Table S3. Alkaloids and polyketides retrieved from *Nocardia* sp.

**Compound**	**Structure, chemical formula and MWT**	**Producing Organisms**	**Biological Activity**	**Reference**
Nocarbenzoxazole D	$C_{15}H_{12}N_2O_4$<br>Mwt: 284.27 g/mol<br>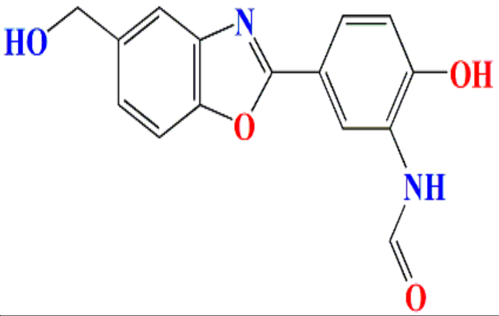 	*Nocardiopsis lucentensis* DSM 44048	Cytotoxic activity	[213]
Questiomycin C	$C_{13}H_{10}N_2O_3S$<br>Mwt: 274.30 g/mol<br>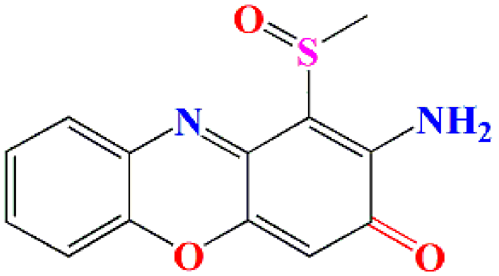 	*Alteromonas sp.*	Algicidal agents	[217]
Questiomycin A	$C_{12}H_8N_2O_2$<br>Mwt: 212.208 g/mol<br>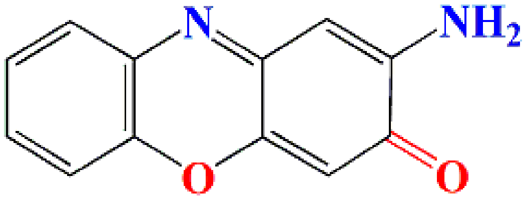 	*N. dassonvillei* JS106	Antiquorum sensing activities	[215]
Nocapyrone E	$C_{12}H_{16}O_2$<br>Mwt: 192.25 g/mol<br>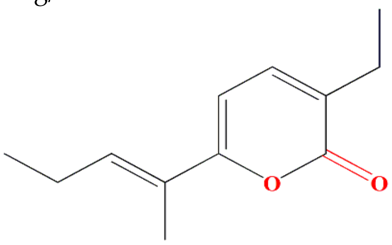 	*Nocardiopsis dassonvillei*	Antimicrobial activity against *Bacillus subtilis*	[218]
(10R)-nocapyrone L	$C_{16}H_{26}O_3$<br>Mwt: 266.38 g/mol	*Nocardiopsis sp.*	-	[219]

	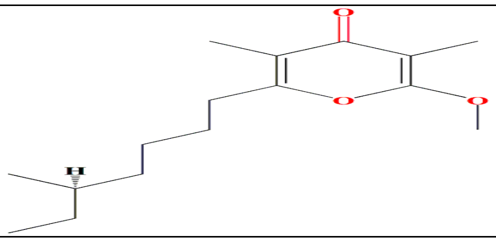			

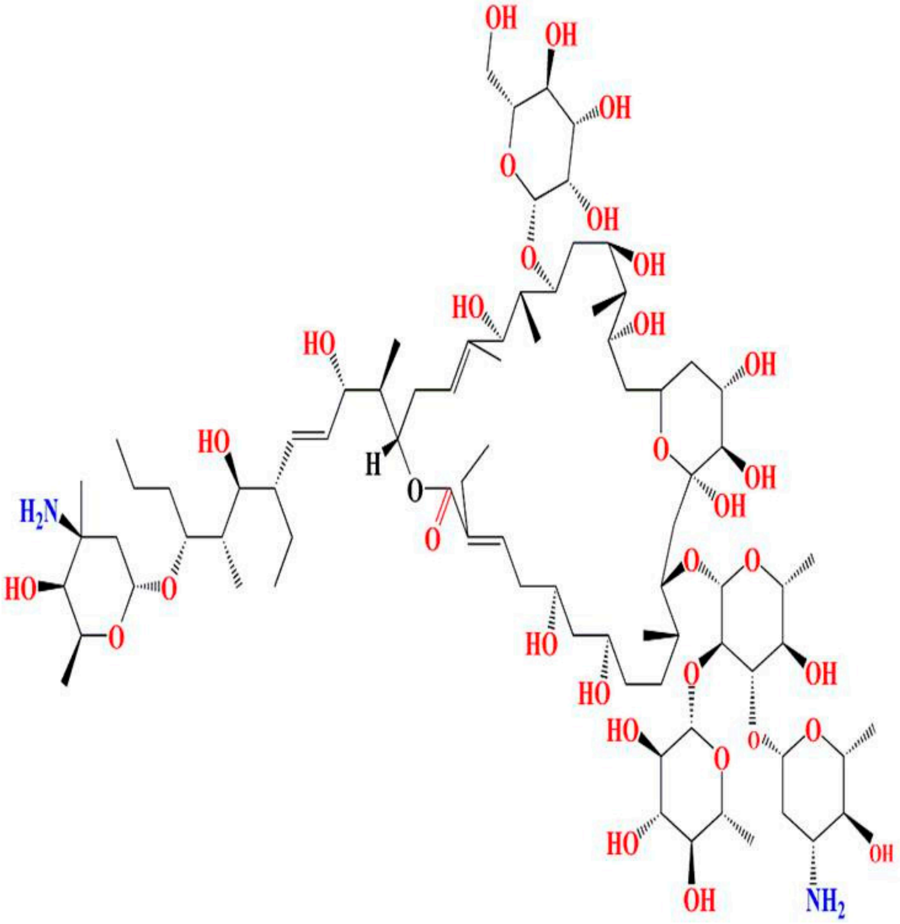

Aculeximycin
$C_{81}H_{144}N_2O_{33}$
1674.0 g/mol

Figure S1. Aculeximycin retrieved from *Kutzneria albida*
